# Social Determinants and Outbreak Dynamics of the 2025 Measles Epidemic in Mexico: A Nationwide Analysis of Linked Surveillance Data

**DOI:** 10.3390/v18020219

**Published:** 2026-02-08

**Authors:** Judith Carolina De Arcos-Jiménez, Pedro Martínez-Ayala, Oscar Francisco Fernández-Diaz, Sergio Sánchez-Enríquez, Patricia Noemi Vargas-Becerra, Ana María López-Yáñez, Roberto Damian-Negrete, Sofía Gutierrez-Perez, Jaime Briseno-Ramírez

**Affiliations:** 1 División Salud, Centro Universitario de Tlajomulco, Universidad de Guadalajara, Tlajomulco de Zuñiga 45641, Mexico; judith.dearcos@academicos.udg.mx (J.C.D.A.-J.); pedro.martinez@cucs.udg.mx (P.M.-A.); oscar.fernandezdiaz@academicos.udg.mx (O.F.F.-D.); patricia.vargas@cutlajomulco.udg.mx (P.N.V.-B.); ana.lopez@academicos.udg.mx (A.M.L.-Y.); roberto.damian@cutlajomulco.udg.mx (R.D.-N.); 2 División de Ciencias Biomedicas, Centro Universitario de Los Altos, Universidad de Guadalajara, Tepatitlán de Morelos 47600, Mexico; sergio.enriquez@cualtos.udg.mx; 3 Comisión Estatal de Derechos Humanos Jalisco, Guadalajara 44600, Mexico; sofiagutierrezperez1982@gmail.com; 4 Hospital Civil de Oriente, Tónala 45425, Mexico

**Keywords:** measles, outbreak investigation, social determinants of health, vaccine effectiveness, molecular epidemiology, spatial analysis, indigenous populations

## Abstract

Measles resurgence threatens elimination achievements in the Americas. We conducted a nationwide analysis of Mexico’s 2025–2026 measles outbreak, integrating individual-level surveillance data from the Special Surveillance System for Febrile Exanthematous Diseases with municipal-level social determinants from eight national databases, complemented by molecular surveillance data. We analyzed 6892 confirmed cases using spatial autocorrelation (Moran’s I and LISA), effective reproduction number estimation, logistic regression models for municipal case presence, and multivariable logistic regression for risk factors for complications. Cases concentrated in Chihuahua (65.2%), with 47 LISA hot-spot municipalities containing 64.4% of cases. Molecular surveillance confirmed two independent introductions: D8/MVs/Ontario.CAN/47.24 (98.1%), linked to the North American outbreak, and B3 (1.9%) in Oaxaca. Transmission followed a three-stage pattern: introduction through seasonal agricultural worker networks, amplification in undervaccinated communities, and diffusion to marginalized indigenous populations. A dual-model analysis revealed that school non-attendance among children aged 6–14 years may have mediated the effect of very high marginalization on municipal case presence (OR 1.26; *p* < 0.001), identifying a potentially actionable vaccination pathway. Vaccine effectiveness was 98.1%, confirming susceptible accumulation rather than vaccine failure. Wave-stratified analysis showed late outbreak phase as an independent risk factor for complications (aOR 1.68, 95% CI: 1.42–2.00), converging with an age of <1 year (aOR 3.36), indigenous status (aOR 1.89), and unvaccinated status (aOR 1.96) in the most marginalized communities. Indigenous individuals comprised 29.1% of cases but 76% of the 25 deaths. This outbreak demonstrates that national vaccination thresholds are insufficient when municipal pockets of susceptibility remain systematically underserved.

## 1. Introduction

Measles remains a significant global public health concern due to its high transmissibility and disease burden, particularly among children in low- and middle-income countries [1,2]. Despite a highly effective vaccine available for over five decades, periodic resurgences persist due to immunization gaps and health system disruptions [3,4].

In 2024, 395,521 laboratory-confirmed cases were reported globally, with 16,147 additional cases in early 2025—likely underestimating true burden due to surveillance limitations [1,5]. Over half required hospitalization, reflecting severe complications, including pneumonia and encephalitis, among unvaccinated and immunocompromised individuals [1,5]. Children under 5 years face the highest burden, with mortality amplified by measles-induced immune amnesia that increases susceptibility to other infections for months to years post-recovery [6,7,8]. Large outbreaks incur substantial economic costs—projected at US$90 million for 2025—while vaccination programs yield an estimated 58:1 return on investment through averted healthcare expenditures and productivity losses [9,10].

Global measles incidence and mortality declined over 90% between 1990 and 2021 through widespread vaccination [5]. Nevertheless, in 2021, measles caused approximately 4.1 million cases, 48,100 deaths, and 4.2 million disability-adjusted life years among children aged <5 years [5]. Burden remains disproportionately concentrated in regions with low sociodemographic indices and suboptimal coverage, such as sub-Saharan Africa and South Asia [5,11,12].

With a basic reproduction number (R_0_) of 12–18, measles requires ≥95% two-dose coverage to prevent outbreaks [3,13]. However, global first-dose (MCV1) coverage stagnated at 83% in 2022–2023—exacerbated by COVID-19 disruptions—with only 64% coverage in low-income countries [1,5,14]. These gaps fueled major outbreaks in 2019 and ongoing crises in 2024–2025, including Europe’s highest case counts in over 25 years and a large outbreak across Texas and New Mexico driven by undervaccinated communities [1,15,16,17].

In the Americas, 18 countries experienced outbreaks between 2019–2023, temporarily disrupting elimination status in two nations; however, no country had endemic transmission by end-2023 [18,19]. Mexico interrupted endemic transmission in 1997 through high coverage and robust surveillance, with subsequent cases primarily importation-driven [20,21].

In post-elimination settings, resurgence is increasingly driven by subnational “pockets of susceptibility”—geographic and social clusters where structural determinants (inequitable access, rurality, marginalization, and mobility networks) concentrate low immunity despite high national coverage [22,23,24,25]. This study provides a comprehensive analysis of Mexico’s 2025 measles epidemic, integrating individual-level surveillance and immunization registry data with municipality-level sociodemographic indicators. We characterize outbreak dynamics, identify high-risk populations, elucidate social determinants driving resurgence, and generate evidence to guide targeted interventions in post-elimination settings.

## 2. Materials and Methods

### 2.1. Study Design and Setting

We conducted a population-based retrospective observational study of Mexico’s 2025–2026 measles epidemic from 19 February 2025 to 18 January 2026 (epidemiological weeks 8–53 of 2025 and weeks 1–3 of 2026). The outbreak included an initial wave (February–December 2025) and an early resurgence in January 2026. Analyses were performed at the individual (confirmed cases) and ecological (municipal) levels across all 32 states and 2469 municipalities.

### 2.2. Data Sources

We integrated eight national databases spanning epidemiological surveillance, hospital discharges, socioeconomic marginalization, migration, agricultural labor, and vaccination coverage. Additional analytic variables were derived a priori based on biological plausibility, including state-relative outbreak week, epidemic phase, binary indicators for high-marginalization and high-migration municipalities, and municipal vaccination gap estimates (data dictionary in Supplementary Table S1).

#### 2.2.1. Primary Data Source: Epidemiological Surveillance

Case-level data were obtained from Mexico’s Special Surveillance System for Febrile Exanthematous Diseases (EFE), maintained by the General Directorate of Epidemiology (DGE) [26]. This open-access database includes 24 clinical–epidemiological variables per case (demographics, clinical manifestations, vaccination and indigenous status, and diagnostic classification) updated weekly through epidemiological week 3 of 2026. Cases were defined per Mexican Official Standard NOM-017-SSA2-2012 [27] and InDRE laboratory guidelines [28] as: (a) laboratory-confirmed (measles-specific IgM or RT-PCR positive) or (b) clinically–epidemiologically confirmed (compatible illness epidemiologically linked to a laboratory-confirmed case). Only confirmed cases were analyzed; suspected, discarded, and rubella cases were excluded.

#### 2.2.2. Hospital Discharge Data

Hospitalization records (ICD-10 code B05) were obtained from the Automated Hospital Discharge Subsystem (SAEH), maintained by the General Directorate of Health Information (DGIS) [29], capturing diagnosis codes, complications, length of stay, and discharge status from Ministry of Health facilities.

#### 2.2.3. Sociodemographic and Marginalization Data

Municipal-level socioeconomic data were obtained from complementary sources: Marginalization Index 2020 (CONAPO) [30]: composite index incorporating nine social deprivation indicators (illiteracy, incomplete basic education, inadequate housing, overcrowding, income below minimum wage, and rurality); Social Lag Index 2020 (CONEVAL) [31]: municipal-level index based on education, health access, housing quality, and basic services; Migration Intensity Index 2020 (CONAPO) [32]: based on households with U.S. emigrants, remittances, circular migrants, and return migrants, characterizing international migration activity; and Indigenous population: proportion self-identifying as indigenous from EFE individual records and CONAPO estimates [26,30].

#### 2.2.4. Agricultural Census Data

To identify municipalities with high agricultural migrant worker activity, we analyzed the 2022 Agricultural Census (INEGI) [33], using variables on agricultural labor force and seasonal worker presence.

#### 2.2.5. Vaccination Coverage Data

Historical vaccination coverage estimates by state and year, including first and second MMR doses, were obtained from the National Center for Child and Adolescent Health (CENSIA) BIO-SIS database (1990–2023; latest state-level annual data were available at time of analysis) [34]. BIO-SIS database coverage data are administrative estimates from CENSIA and subject to the inherent limitations of this data source.

#### 2.2.6. Molecular Surveillance Data

Measles virus genomic sequencing data were obtained from GenBank (NCBI) [35], yielding 26 historical N-450 nucleoprotein gene sequences from Mexico (2003–2021) and from Epidemiological Bulletins (DGE) [36], providing 2025 outbreak data aggregated by state (number of sequences, genotype, and lineage) through epidemiological week 48 of 2025.

#### 2.2.7. Population Denominators

Population denominators by municipality and age group were obtained from CONAPO mid-year 2025 projections [37] for calculating age- and municipality-specific incidence rates throughout the 2025–2026 outbreak period.

### 2.3. Data Linkage

All databases were linked at the municipal level using the official five-digit INEGI geographic code (clave geoestadística), consisting of two-digit state and three-digit municipality identifiers. This standardized coding enabled deterministic linkage across all sources. Complete socioeconomic indicators were available for 99.5% of municipalities (n = 2457).

### 2.4. Statistical Analysis

#### 2.4.1. Descriptive Analysis

We characterized the epidemic through temporal (epidemic curves by epidemiological week), geographic (state and municipality distribution), and demographic (age, sex, indigenous status, and vaccination status) dimensions. Incidence rates were calculated per 100,000 population using CONAPO 2025 projections as denominators: case fatality rates as deaths divided by confirmed cases [37].

Epidemic phase classification. Two temporal frameworks were employed: (1) absolute epidemiological weeks for national-level summaries and (2) state-relative weeks (from each state’s index case) for social determinants analysis. Relative phases were: introduction (weeks 1–4), growth (weeks 5–11), peak (weeks 12–14), decline (weeks 15–24), and late (weeks 25+). Cases from the January 2026 resurgence (weeks 54–56) were classified separately as “Resurgence (Wave 2)” to distinguish them from the primary outbreak. National absolute phases were: Introduction (weeks 8–16), Peak (weeks 17–28), Decline (weeks 29–40), Late (weeks 41–53), and Resurgence (weeks 54–56).

#### 2.4.2. Transmission Dynamics

The time-varying effective reproduction number (Rt) was estimated using Bayesian inference with a gamma-distributed serial interval (mean 11.7 days, SD 2.0 days) derived from measles contact-tracing studies [38]. Rt was calculated with a 7-day sliding window, reporting posterior means and 95% credible intervals (CrI) at each time point. National Rt was estimated from aggregated daily case counts, and state-level Rt was computed for the six highest-burden states (Chihuahua, Jalisco, Chiapas, Guerrero, Michoacán, Sinaloa). Sustained transmission was defined as Rt > 1. Estimates were obtained using the EpiEstim R package, which implements a Bayesian framework to infer the instantaneous reproduction number from incidence data while incorporating the serial interval distribution and its uncertainty [39,40]. Interpretation follows standard thresholds: Rt > 1 indicates exponential growth, Rt = 1 stable transmission, and Rt < 1 declining transmission. Additional methodological details are provided in the Supplementary Materials (Supplementary Note S1).

#### 2.4.3. Spatial Analysis

Spatial autocorrelation of measles incidence was assessed at the municipal level (n = 2457) using exploratory spatial data analysis. Incidence rates per 100,000 were calculated using CONAPO 2025 population projections [37]. A spatial weights matrix was constructed using queen contiguity with row-standardized weights. Global spatial autocorrelation was evaluated using Moran’s I statistic [41], with significance assessed through conditional permutation (*p* < 0.05).

Local Indicators of Spatial Association (LISA) were calculated using local Moran’s I [42] to identify significant clusters (*p* < 0.05): hot spots (High-High: high incidence surrounded by high incidence), cold spots (Low-Low), and spatial outliers (High-Low or Low-High). To capture changes in clustering across epidemic phases, LISA was estimated separately for Wave 1 (2025) and Wave 2 (January 2026), and within individual states with ≥100 cases to detect intra-state patterns not visible at the national scale. To assess temporal–spatial spread, we calculated the Euclidean distance from each affected municipality to the outbreak epicenter (Cuauhtémoc, Chihuahua) and examined its relationship with case detection timing using linear regression. The epicenter was defined as the municipality reporting the earliest sustained case cluster. A detailed description of the mathematical formulation, spatial weight specification, and multi-level analytical approach is provided in the Supplementary Materials [43,44] (Supplementary Figure S1).

#### 2.4.4. Social Determinants and Introduction Mechanism

We conducted a municipal-level ecological analysis comparing 266 municipalities with confirmed cases versus 2203 without cases, linking municipal indicators (by residence municipality) from four national sources: the CONAPO Marginalization Index 2020 (0–100; degree categories) [30], CONAPO Migration Intensity Index 2020 (null to very high) [32], CONEVAL Social Lag Index 2020 (including proportion without health insurance) [31], and INEGI 2022 Agricultural Census (percentage employing seasonal workers) [33]. These indicators reflect municipal context rather than individual attributes. Bivariate comparisons used Mann–Whitney U tests for continuous variables and chi-squared tests for categorical variables. Multivariable analyses used negative binomial regression of case counts with log(population) as an offset, reporting incidence rate ratios (IRRs) with 95% CIs. Analyses were stratified by (1) outbreak phase using state-relative weeks (introduction, 1–4; growth, 5–11; peak, 12–14; decline, 15–24; late, 25+; resurgence, January 2026), (2) the six states with ≥50 cases (95.5% of total), and (3) a sensitivity comparison of the first vs. last 50 affected municipalities chronologically. Temporal trends were assessed using Spearman rank correlation and LOESS smoothing (span = 0.4) with 95% CIs to visualize non-linear patterns.

#### 2.4.5. Vaccination and Case Incidence

Vaccine effectiveness (VE) was estimated using the Farrington screening method [45]: VE = 1 − [(PCV × (1 − PPV))/(PPV × (1 − PCV))], and VE = 1 − [(PCV × (1 − PPV))/(PPV × (1 − PCV))], where PCV is the proportion of cases vaccinated (EFE data), and PPV is the population proportion vaccinated (mean MCV1 coverage 2014–2023, CENSIA/CNI). Ninety-five percent CIs were calculated using the Orenstein method [46,47]. State-level VE was estimated for states with ≥20 cases using state-specific coverage. Additional metrics included relative risk (RR) and population attributable fraction (PAF). To characterize focal transmission, municipalities with ≥10 cases were classified by the proportion of unvaccinated cases (<70%, 70–79%, 80–89%, 90–94%, and ≥95%) to identify “pockets of susceptibles” where transmission concentrated. Further details on VE estimation and interpretation in outbreak settings are provided in the Supplementary Materials (Supplementary Figure S2), consistent with prior outbreak investigations and immunization guidelines [48,49,50,51].

#### 2.4.6. Complications and Risk Factors for Severity

Risk factors for complications (pneumonia, otitis media, encephalitis, and others) were assessed using logistic regression. Individual-level covariates included age group (<1, 1–4, 5–19, and ≥20 years), sex, vaccination status, and indigenous self-identification. Municipal-level contextual variables included marginalization degree; rurality (>50% of the population living in localities of <5000); and access to piped water, drainage, and health services (CONAPO 2020 [30] and CONEVAL 2020 [31]). All plausible predictors were screened in bivariable analyses; variables with *p* < 0.20 were considered for multivariable modeling [52,53]. The final model prioritized parsimony, control of collinearity (VIF < 5), and model fit (AIC, pseudo-R^2^). Results are reported as crude (OR) and adjusted odds ratios (aOR) with 95% CIs. Due to the low number of deaths (n = 25), mortality analyses were descriptive only.

#### 2.4.7. Molecular Epidemiology

Molecular analysis was based on 207 sequences; data for weeks 49–56 (through January 2026) were unavailable. Spatial autocorrelation of the week of symptom onset was assessed using Moran’s I with a k = 4 nearest-neighbor spatial weight matrix. Linear regression fitted the distance from the epicenter (Chihuahua) as the predictor and the week of first case as the outcome to estimate propagation speed. Distances were calculated using the Haversine formula, with speed estimated as a distance/time ratio, stratified by region (North, Central, and South). States with non-predominant genotypes were evaluated separately to identify independent introductions.

#### 2.4.8. Software and Reproducibility

All analyses used R version 4.4.1. Data management and visualization: tidyverse (v2.0.0), ggplot2 (v4.0.1), and dplyr (v1.1.4); spatial analysis: sf (v1.0.23) and spdep (v1.3.13); Rt estimation: EpiEstim (v2.2.5); additional packages: broom (v1.0.9), gtsummary (v2.3.0), flextable (v0.9.9), and patchwork (v1.3.2). Scripts are available in a reproducible pipeline with centralized configurations, enabling re-execution with updated data, upon request to the corresponding author.

## 3. Results

### 3.1. Descriptive Analysis

**Figure 1:**
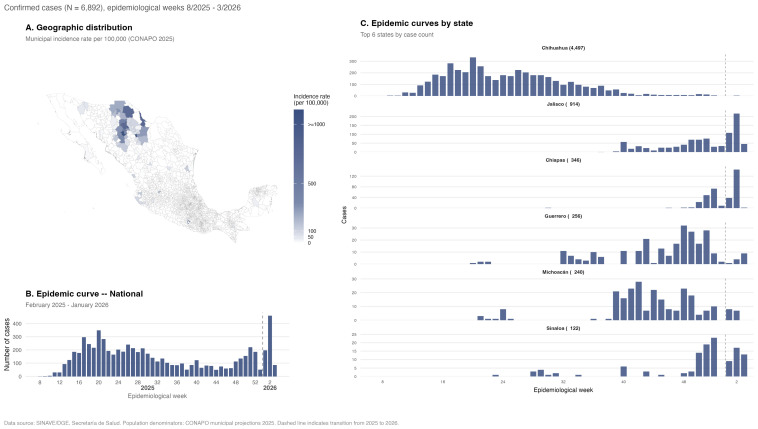
Measles outbreak overview—in Mexico—for epidemiological weeks August 2025–March 2026. (**A**) Geographic distribution of confirmed cases showing municipal incidence rates per 100,000 population (CONAPO 2025 projections [37]). (**B**) National epidemic curve, with the dashed line indicating 2025–2026 transition. (**C**) State-level epidemic curves for the six most affected states.

A total of 6892 confirmed measles cases (6432 laboratory-confirmed; 460 epidemiologically linked) were reported across all 32 states during epidemiological weeks 8–53/2025 and 1–3/2026, with marked geographic concentration in northern Mexico (Figure 1).

Chihuahua accounted for 65.2% of cases (n = 4497). Cases in Chihuahua were older (median 20 years, IQR 4–31 vs. 12 years, IQR 4–24; *p* < 0.001), with adults of 20–39 years comprising 41.5% of the population versus 27.8% in other states (Table 1). Overall, 85.5% of cases were unvaccinated, and 29.1% were indigenous. Import-related transmission predominated in Chihuahua (78.2% vs. 20.0% in other states; *p* < 0.001). Complications occurred in 15.5% of cases, significantly higher in Chihuahua (19.1% vs. 8.7%; *p* < 0.001). Twenty-five deaths were recorded (CFR 0.4%), predominantly in Chihuahua (n = 23; *p* = 0.009).

Cases with complications (n = 1069, 15.5%) were younger (median 4 years, IQR 1–18 vs. 19 years; *p* < 0.001), with children <5 years accounting for 51.4% of complications (Supplementary Table S2). Indigenous individuals represented 51.3% of complicated cases versus 25.0% of uncomplicated cases (*p* < 0.001) (Table S3).

Five outbreak phases were identified: Introduction (weeks 8–16, n = 647), Peak (17–28, n = 2770), Decline (29–40, n = 1398), Late (41–53, n = 1336), and Resurgence (54–56, n = 741) (Supplementary Table S4). The most notable temporal shift was in indigenous status, rising from 0.8% during Introduction to 54.6% during Decline (*p* < 0.001). Complication rates peaked during Decline (25.1%), and deaths occurred primarily during Peak (n = 10) and Decline (n = 12, CFR 0.9%).

### 3.2. Transmission Dynamics

**Figure 2:**
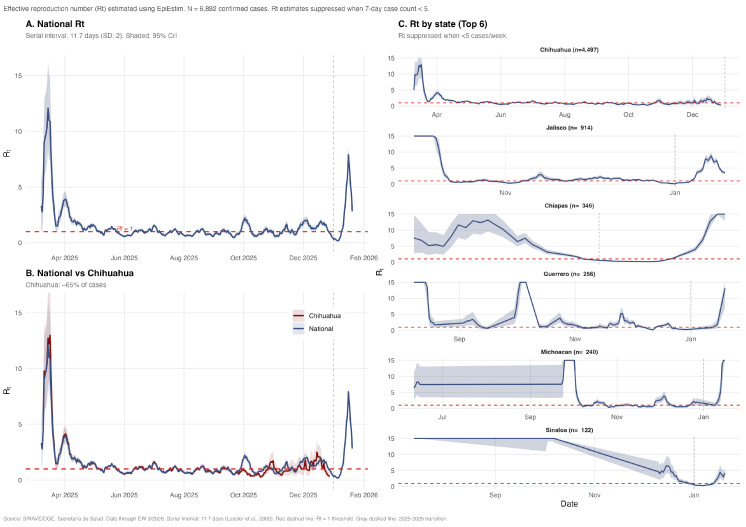
Effective reproduction number (Rt) dynamics during the measles outbreak—Mexico, 2025. (**A**) National Rt estimates showing peak transmission in mid-March and subsequent decline below the epidemic threshold (dashed red line, Rt = 1). (**B**) Comparison of national and Chihuahua-specific Rt trajectories, demonstrating the dominant contribution of Chihuahua to overall epidemic dynamics. (**C**) State-level Rt estimates for the five most affected states, showing distinct temporal patterns of outbreak introduction and transmission. Shaded areas represent 95% credible intervals. Serial interval assumed at 11.7 days (SD: 2.0 days) [38].

The national effective reproduction number peaked at 12.1 (95% CrI: 8.5–16.4) on 15 March 2025 during the early growth phase, indicating intense initial transmission (Figure 2A). Rt declined progressively, crossing below 1 on 8 May, approximately 8 weeks after the peak. National Rt remained below 1 from May through December 2025, consistent with controlled transmission during the Decline and Late phases. In Chihuahua (~65% of cases), the Rt trajectory closely paralleled national estimates, peaking at 13.0 on 17 March (Figure 2B). Chihuahua’s Rt first declined below 1 on 6 May, and the last reliable estimate was 0.38 (28 December 2025), indicating controlled transmission in the epicenter by year-end.

State-level Rt estimates showed distinct temporal patterns reflecting heterogeneous outbreak timing (Figure 2C). States with a later epidemic onset (Jalisco, Chiapas, Guerrero, and Michoacan) exhibited elevated Rt values during their respective introduction phases, with wider credible intervals due to smaller case counts. By January 2026, the national Rt had risen to 2.81, driven by the resurgence wave predominantly affecting states outside Chihuahua. Table S5 reports state-specific exact values (peak Rt, peak dates, number of days with Rt > 1, and last status) and the extended methodology for reference.

### 3.3. Spatial Analysis

**Figure 3:**
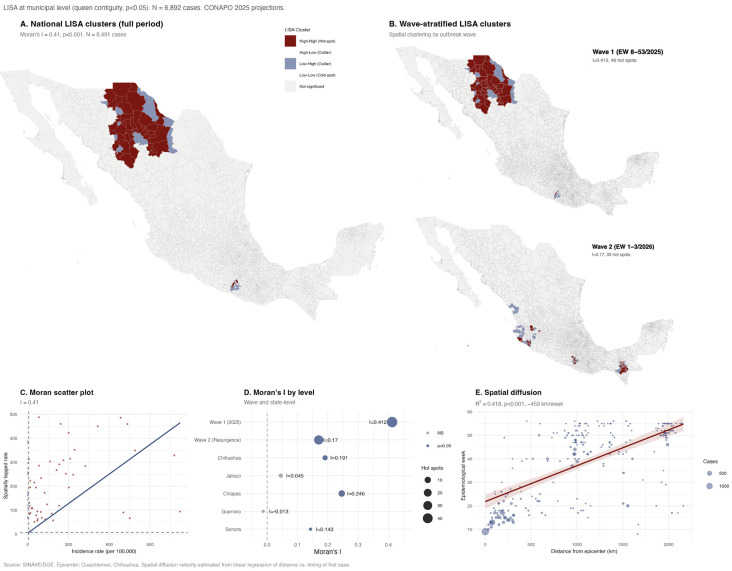
Spatial analysis of measles outbreak—Mexico, 2025–2026. (**A**) National LISA cluster classification at municipal level; hot spots (High-High) are in red, and cold spots (Low-Low) in blue. (**B**) Wave-stratified LISA comparing Wave 1 (EW 8–53/2025) and Wave 2 (EW 1–3/2026), showing a shift from concentrated to dispersed clustering. (**C**) Moran scatter plot of municipal incidence rate versus spatially lagged rate. (**D**) Comparison of Moran’s I across analytical levels (wave-stratified and state-level). (**E**) Spatial diffusion pattern: relationship between distance from the epicenter and epidemiological week of first case arrival.

Measles incidence showed strong national spatial autocorrelation (Moran’s I = 0.41, z = 35.3; *p* < 0.001), confirming marked geographic clustering (Figure 3A). LISA identified 47 hot-spot municipalities—predominantly in Chihuahua—accounting for 4441 cases, with highest incidence in Sierra Tarahumara areas characterized by large indigenous populations and limited healthcare access. Spatial clustering shifted across epidemic phases (Figure 3B): Wave 1 (2025) was highly localized (I = 0.412; 46 hot spots, mostly in Chihuahua), whereas during the Wave 2 resurgence (January 2026), clustering weakened (I = 0.17) and dispersed across multiple states (33 hot spots) (Supplementary Table S6). State-level analyses revealed significant intra-state clustering in Chiapas, Chihuahua, and Sonora, while Jalisco and Guerrero showed no spatial autocorrelation, consistent with metropolitan rather than contiguous spread (Figure 3D; Supplementary Figure S1). Distance from the outbreak epicenter (Cuauhtémoc, Chihuahua) was strongly associated with the timing of first case arrival (R^2^ = 0.418; *p* < 0.001), supporting radial diffusion at approximately 459 km/week through population mobility networks (Figure 3E).

### 3.4. Social Determinants and Introduction Mechanism

**Figure 4:**
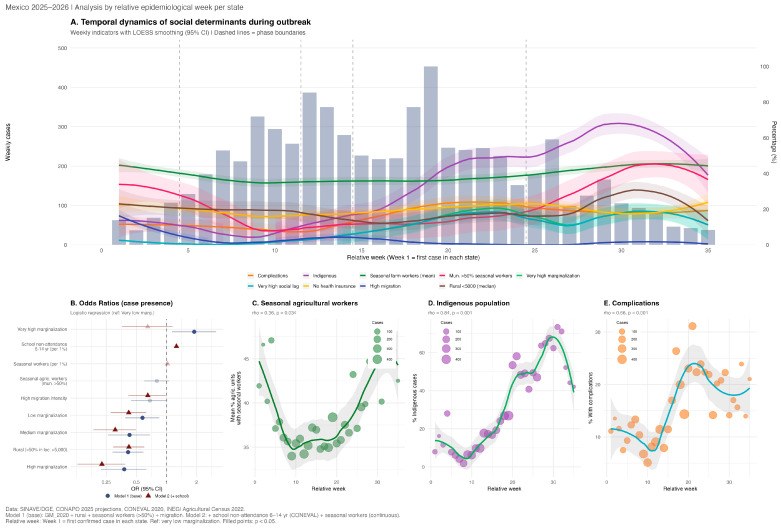
Social determinants of measles incidence during the outbreak in Mexico in 2025–2026 [26,31,33,37]. (**A**) Weekly epidemic curve (bars) overlaid with LOESS-smoothed trends for nine municipal-level social indicators by relative week (Week 1 = first confirmed case in each state). Dashed vertical lines indicate outbreak phase boundaries (Introduction, Growth, Peak, Decline, and Late). (**B**) Odds ratios (OR) from two multivariable logistic regression models for municipal case presence. Model 1 (blue circles): base model with marginalization degree, rurality, seasonal agricultural workers, and migration intensity. Model 2 (red triangles): adds school non-attendance among children aged 6–14 years (CONEVAL) and seasonal workers (continuous). Faded points indicate *p* ≥ 0.05. (**C**) Mean percentage of agricultural production units employing seasonal agricultural workers by relative week (Spearman rho = 0.36; *p* = 0.034). (**D**) Percentage of indigenous cases by relative week (Spearman rho = 0.84; *p* < 0.001). (**E**) Percentage of cases with complications by relative week (Spearman rho = 0.66; *p* < 0.001).

Municipalities with confirmed measles cases (n = 266, 10.4%) differed significantly from those without cases across multiple social indicators (Table 2). Affected municipalities had lower rurality (median 19.3% vs. 48.8%; *p* < 0.001) and a bimodal marginalization distribution—with overrepresentation of both very low and very high categories. In a base logistic regression for case presence (Model 1, Figure 4B), only very high marginalization was positively associated with having cases (OR 1.89, 95% CI 1.13–3.14; *p* = 0.014), while low-, medium-, and high-marginalization categories were less likely affected than the very low (urban) reference (OR 0.38–0.58; all *p* < 0.01), and rural municipalities showed lower odds (OR 0.41, 95% CI 0.29–0.59; *p* < 0.001). When school non-attendance among children aged 6–14 years (CONEVAL) was added to the model (Model 2), it emerged as the strongest predictor of municipal case presence (OR 1.26 per 1% increase, 95% CI 1.21–1.31; *p* < 0.001), while very high marginalization lost significance (OR 0.65; *p* = 0.14), suggesting that vaccination gaps in communities where children do not attend school mediate the marginalization effect (Table S7). Model 2 showed a substantially improved fit (AIC 1380 vs. 1522; AUC 0.795 vs. 0.716). The inverse association with migration intensity (OR 0.65, 95% CI 0.42–0.98; *p* = 0.049) reflects that the CONAPO index measures emigration to the USA—distinct from the cross-border agricultural worker mobility that introduced measles into northern Mexico.

Temporal analysis by outbreak phase revealed pronounced shifts in the social profile of cases (Table 3; Figure 4). Early transmission (weeks 1–4; n = 274) was concentrated in periurban areas, with municipalities comprising high seasonal agricultural workers (40.4%) and with fewer indigenous cases (17.9%) and complications (10.2%). By the late phase (weeks ≥ 25; n = 1080), spread had shifted to remote, marginalized communities, with indigenous cases rising to 65.8%, complications to 23.1%, and median age decreasing from 22 to 11 years, indicating involvement of younger, unvaccinated cohorts (Figure 4C–E). The share of cases from high agricultural seasonal worker municipalities followed a U-shaped pattern—declining to 7.1% at peak and rebounding to 29.9% at late outbreak—suggesting seasonal re-activation of agricultural labor networks.

State-level stratification (Table S8) confirmed heterogeneous social profiles: Chihuahua (4497 cases; 73.4% average vaccine coverage) was characterized by periurban transmission, while Guerrero (256 cases) and Chiapas (346 cases) showed predominantly indigenous and rural case profiles. Sensitivity analysis comparing the first and last 50 municipalities by temporal sequence (Table S9) reinforced the introduction-to-dispersion gradient: early-affected municipalities had an older median age, lower indigenous proportion, and lower seasonal worker concentration than late-affected municipalities. State-level vaccine coverage (2019–2023 average) was lower in states with ≥100 confirmed cases (mean, 80.8%) compared to states with fewer or no cases (mean 86.5%), though Chihuahua exhibited the lowest coverage among all affected states (73.4%), consistent with long-standing vaccination gaps (Table S8).

### 3.5. Vaccination and Vaccine Effectiveness (VE)

**Figure 5:**
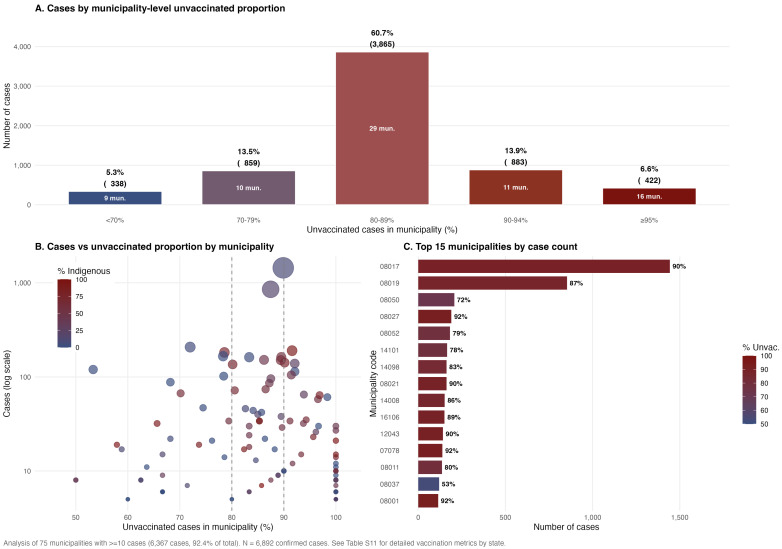
Municipality-level vaccination gaps and pockets of susceptibles during the measles outbreak in Mexico, 2025–2026. (**A**) Distribution of confirmed cases by municipality-level proportion of unvaccinated cases; 60.7% of cases (3865) concentrated in 29 municipalities where 80–89% of cases were unvaccinated. (**B**) Scatter plot of municipality case count (log scale) versus proportion of unvaccinated cases, colored by percentage of indigenous population; dashed vertical lines indicate 80% and 90% thresholds. (**C**) Top 15 municipalities by case count with horizontal bars colored by proportion of unvaccinated cases. Analysis restricted to 75 municipalities with ≥10 cases (6367 cases; 92.4% of total). n = 6892 confirmed cases. See Table S11 for detailed vaccination metrics by state.

Among 6892 confirmed cases, 5895 (85.5%) were unvaccinated. National vaccine effectiveness estimated by the Farrington screening method (Supplementary Figure S2) was 98.1% (95% CI: 98.0–98.2%), with a PCV of 14.5% and population vaccination coverage (PPV) of approximately 90% (Supplementary Figure S2B, Table S10). VE ranged from 93.5% in Jalisco to 99.1% in Guerrero among states with ≥50 cases (Table S9). The relative risk for unvaccinated individuals was 53.2, and the population attributable fraction was 97.8%, indicating that an estimated 6741 of 6892 cases were attributable to lack of vaccination. Municipality-level analysis revealed pronounced pockets of susceptibles: among 75 municipalities with ≥10 cases (representing 92.4% of all cases), 60.7% of cases (3865) concentrated in 29 municipalities where 80–89% of cases were unvaccinated, and an additional 20.5% in municipalities with ≥90% unvaccinated (Figure 5A). The two highest-burden municipalities (Cuauhtemoc and Guachochi, Chihuahua) each exceeded 1000 cases with 87–90% unvaccinated, and municipalities with the highest indigenous population proportions showed the most extreme vaccination gaps (Figure 5B,C).

State-level MCV1 coverage (2014–2023 average) showed no significant correlation with 2025 incidence rates (Spearman rho = −0.13; *p* = 0.46), consistent with the focal, sub-state nature of the outbreak. Chihuahua had the lowest mean historical coverage (77.6%), with those 15 to 34 years below 80% and a 2023 coverage of only 65.6%—far below the 95% herd immunity threshold (Supplementary Figure S2A). Proportions of vaccinated cases were lowest among infants < 1 year (5.4%), reflecting a pre-vaccination age, and highest among ages 1–4 (16.4%) and 20–39 years (16.2%), suggesting immunity gaps in these cohorts (Supplementary Figure S2C). Birth-cohort analysis revealed case accumulation across cohorts born between 1993 and 2022, with peaks among children born in 2020–2022 (ages 3–5) and adults born in 1993–2000 (ages 25–32), corresponding to periods of lower national MCV1 coverage in the late 1990s (Supplementary Figure S2D). The absence of a correlation between cohort-level coverage and case counts (rho = 0.17; *p* = 0.36) underscores that susceptibility accumulated through multiple mechanisms beyond routine immunization failures, including missed booster doses and waning immunity in adults.

### 3.6. Complications and Risk Factors for Severity

Overall, 1069 of 6892 cases (15.5%) developed complications. In the bivariable analysis, 26 of 28 variables tested were associated with complications at *p* < 0.20 (Table S12). The multivariable model (Table 4) identified six independent risk factors. Age < 1 year carried the highest risk (aOR 3.36, 95% CI 2.72–4.15), followed by ages 1–4 (aOR 2.58, 2.14–3.13), while adults ≥ 20 years had lower risk (aOR 0.64, 0.53–0.77) compared to the 5–19 reference group. Indigenous status (aOR 1.89, 1.61–2.22), lack of vaccination (aOR 1.96, 1.53–2.51), late outbreak phase (aOR 1.68, 1.42–2.00), and rural municipality of residence (aOR 1.73, 1.48–2.03) were all significantly associated with complications (all *p* < 0.001). The resurgence wave (January 2026) was not independently associated with increased complications (aOR 0.81, 0.60–1.09; *p* = 0.164), despite the overall lower complication rate (8.5% vs. 16.4% in Wave 1), suggesting that observed differences reflect changes in the geographic and demographic composition of cases rather than a secular trend.

Complication rates showed stark gradients: rural (27.5%) vs. urban (12.3%) municipalities, indigenous (27.4%) vs. non-indigenous (10.7%) cases, and late-phase (22.5%) vs. early-phase (9.2%) cases. These gradients converge geographically—as the outbreak migrated from urban periurban areas into remote indigenous communities during late phases (Section 3.4), the cumulative burden of complications concentrated in the most marginalized populations.

#### Hospital Discharge Analysis

Linkage with the Automated Hospital Discharge Subsystem (SAEH) identified 663 measles-coded hospitalizations in Ministry of Health facilities during February–November 2025 (Table S13). Among hospitalized patients, 308 (46.5%) had documented complications, predominantly pneumonia (n = 270, 87.7% of complications), followed by other complications (n = 18, 5.8%), encephalitis (n = 10, 3.2%), and otitis media (n = 3, 1.0%). Pneumonia was most frequent among children aged 5–9 years (49.4%) and 1–4 years (47.9%). Median length of stay was 4 days (IQR: 2–6). Indigenous patients had significantly higher complication rates (50.3% vs. 41.6%; *p* = 0.033) and longer hospitalizations (*p* < 0.001). Six in-hospital deaths occurred (case fatality rate among hospitalized: 0.9%), with three (50%) associated with pneumonia. Twenty-five total deaths were recorded during the epidemic (overall CFR: 0.36%).

### 3.7. Molecular Epidemiology

**Figure 6:**
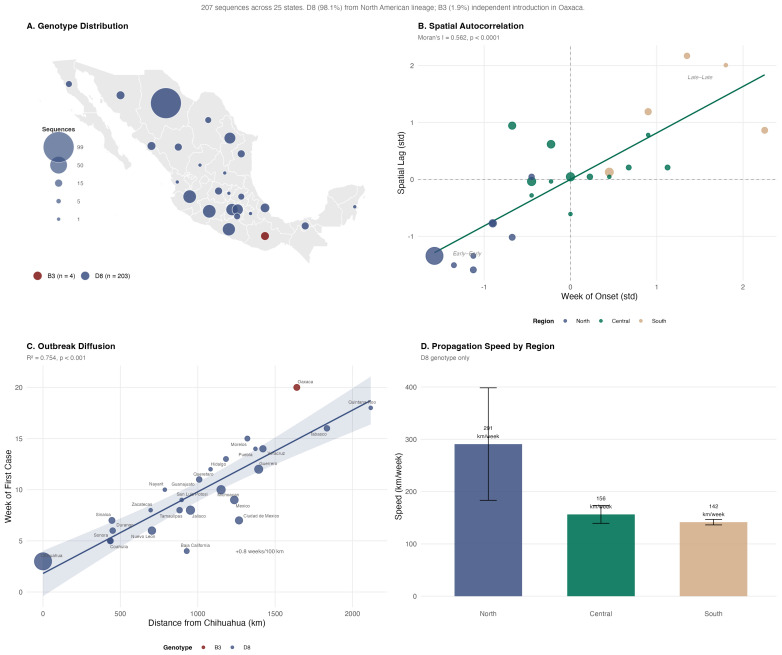
Molecular epidemiology of the 2025 measles outbreak in Mexico. (**A**) Geographic distribution of circulating genotypes: D8 (98.1%) in blue and B3 (1.9%) in red (Oaxaca only). (**B**) Moran scatter plot demonstrating significant spatial autocorrelation of epidemiological week of onset (I = 0.562; *p* < 0.0001), confirming contagious diffusion. (**C**) Linear relationship between distance from epicenter (Chihuahua) and week of first case (R^2^ = 0.754); Oaxaca (B3) appears as an outlier. (**D**) Mean propagation speed by region showing faster spread in the North (291 km/week) compared to Central (156 km/week) and South (142 km/week). The national mean propagation speed for genotype D8 was 194.8 km/week, calculated as a population-weighted average across regions.

Genomic surveillance based on 207 sequences across 25 states (Table S14) identified two co-circulating genotypes. Genotype D8 (lineage MVs/Ontario.CAN/47.24) predominated with 203 sequences (98.1%) in 24 states, while B3 (lineage MVs/New South Wales.AUS/10.24) comprised 4 sequences (1.9%) restricted to Oaxaca, indicating at least two independent introductions. Spatial analysis of onset timing demonstrated significant autocorrelation (Moran’s I = 0.562; *p* < 0.0001), consistent with contagious diffusion from the Chihuahua epicenter (Figure 6A,B). Distance from Chihuahua explained 75.4% of the variance in the week of first case arrival (R^2^ = 0.754; *p* < 0.001), with each 100 km adding approximately 0.80 weeks (5.6 days) to the arrival time (Figure 6C). The mean D8 propagation speed was 194.8 km/week (27.8 km/day), being fastest in the North (290.9 km/week), intermediate in the Center (156.5 km/week), and slowest in the South (141.6 km/week) (Figure 6D). Oaxaca’s B3 genotype arrived 5.1 weeks later than predicted by the D8 diffusion model (observed: week 20 vs. predicted: week 14.9), confirming an independent introduction unrelated to the northern epicenter. Historically, only 26 measles sequences from Mexico were deposited in GenBank (2003–2021), representing sporadic importations of genotypes D4, D8, D9, and H1 (Supplementary Figure S3). The 2025 outbreak represents an unprecedented scale of measles genomic detection in the country.

## 4. Discussion

This nationwide analysis of 6892 confirmed measles cases concerns Mexico’s largest epidemic since interruption of endemic transmission in 1997 and provides comprehensive insight into the outbreak dynamics, social determinants, and molecular origins of this resurgence [20]. Six principal findings emerge. First, the outbreak was highly focal: Chihuahua accounted for 65.2% of cases, and 47 LISA hot-spot municipalities concentrated 64.4% of the national burden. Second, molecular surveillance confirmed two independent introductions—a predominant D8 genotype (98.1%, lineage MVs/Ontario.CAN/47.24) linked to the 2024–2025 North American outbreak, and a separate B3 genotype (1.9%) imported into Oaxaca from Australia. Third, transmission followed a three-stage pattern: introduction through seasonal agricultural worker networks, amplification in undervaccinated communities, and subsequent diffusion toward marginalized indigenous populations. Fourth, a dual-model analysis revealed that school non-attendance among children aged 6–14 years mediated the effect of very high marginalization on municipal case presence, identifying a previously unreported and potentially actionable pathway for targeted vaccination. Fifth, vaccine effectiveness remained high (98.1%), confirming that the outbreak was driven by accumulation of susceptible individuals rather than vaccine failure. Sixth, independent risk factors for complications—age of <1 year (aOR 3.36), age of 1–4 years (aOR 2.58), unvaccinated status (aOR 1.96), indigenous status (aOR 1.89), rural residence (aOR 1.73), and late outbreak phase (aOR 1.68)—converged geographically in the most marginalized communities reached last by the epidemic.

The predominance of D8/MVs/Ontario.CAN/47.24 places Mexico’s outbreak within the broader 2024–2025 North American measles resurgence. The lineage was first characterized in Canada (Ontario, epidemiological week 47 of 2024), but the same genotype circulated across at least eight countries, linked by Mennonite community travel networks [54]. In the United States, 2267 confirmed measles cases were reported in 2025—the highest annual total since elimination was declared in 2000—including a Southwest US outbreak of 933 cases across Texas (646), New Mexico (65), and neighboring states, with three deaths [55,56,57,58]. Mathematical modeling of the Texas–New Mexico cluster estimated effective reproduction numbers of 1.65–3.82 depending on model structure (exponential growth, SIR, or SEIR), with basic reproduction numbers of 30–40 [17]. Our peak national Rt of 12.1 (95% CrI: 8.5–16.4) is not directly comparable, as it represents instantaneous Bayesian estimations (EpiEstim), capturing peak transmission intensity, whereas the Texas–New Mexico estimates reflect growth-phase averages from mechanistic models; nevertheless, both outbreaks confirm above-threshold transmission in undervaccinated communities. Given Chihuahua’s shared border with Texas and New Mexico, and the well-documented cross-border mobility of Mennonite communities between these regions, transmission through the US–Mexico border represents a plausible and likely route of introduction—consistent with, rather than contradicting, the Canadian lineage designation [59]. Notably, the high proportion of import-related cases in Chihuahua (78.2% vs. 20.0% in other states) reflects this extensive inter-municipal and cross-border mobility, as SINAVE classifies any infection acquired outside the municipality of residence as “imported,” encompassing inter-municipal, inter-state, and international movement. Indeed, the temporal and geographic overlap of the Gaines County, Texas, outbreak (January–August 2025) with Mexico’s epidemic (February 2025 onward) and the shared D8 genotype across both countries support a connected North American transmission chain. Collectively, these outbreaks contributed to the loss of measles elimination status in the Region of the Americas, declared by PAHO in November 2025 after Canada documented >12 months of sustained transmission [60]. In January 2026, PAHO invited both the United States and Mexico for a formal review of their individual elimination status [61], underscoring the regional dimension of this resurgence [18].

Spatial analysis at two complementary scales provided evidence of contagious diffusion. At the municipal level, incidence showed strong spatial autocorrelation (Moran’s I = 0.41; *p* < 0.001), with distance from the Cuauhtémoc epicenter explaining 42% of the variance in timing of first case arrival at approximately 459 km/week through population mobility networks. At the state level, molecular surveillance showed even stronger spatial structuring (R^2^ = 0.754), with a propagation speed of 194.8 km/week, reflecting the broader North-to-South diffusion pattern, consistent with contagious rather than strongly hierarchical diffusion [62]. Wave-stratified LISA analysis revealed a transition from concentrated clustering during Wave 1 (I = 0.412, 46 hot spots in Chihuahua) to more dispersed patterns during the January 2026 resurgence (I = 0.17, 33 hot spots across multiple states), consistent with epidemic maturation from focal introduction to wider geographic dissemination.

A novel finding is the identification of school non-attendance as a mediator of the relationship between very high marginalization and municipal case presence. In the base model, very high marginalization was the only category positively associated with case presence (OR 1.89; *p* = 0.014), with a bimodal pattern reflecting simultaneous vulnerability in urban hubs (very low marginalization) and remote indigenous communities. When school non-attendance among children aged 6–14 years was added, it emerged as the strongest predictor (OR 1.26 per 1% increase; *p* < 0.001), while very high marginalization lost significance (OR 0.65; *p* = 0.14), and model fit improved substantially (AIC 1380 vs. 1522; AUC 0.795 vs. 0.716; Table S7). This mediation pattern suggests that the excess risk in very highly marginalized municipalities operates, at least partly, through children not enrolled in or attending school—and therefore not exposed to school-based vaccination campaigns and coverage verification. These findings extend evidence that multiple deprivations drive suboptimal childhood vaccination in Latin America [22] by identifying a specific, actionable indicator that could prioritize municipalities for supplementary vaccination activities.

The progressive concentration of cases among indigenous populations—from 0.8% during introduction to 54.6% during the decline phase (*p* < 0.001)—mirrors patterns observed in Ecuador, where measles odds were fourfold higher in parishes with larger indigenous populations [22]. PAHO reported that most measles deaths in Mexico’s 2025 outbreak occurred among indigenous individuals [63], consistent with our finding that indigenous persons comprised 29.1% of cases but 76% of the 25 deaths. The convergence of indigenous status, young age, lack of vaccination, rural residence, and late outbreak phase in the multivariable model underscores that complications concentrated where the epidemic arrived last: remote communities with multiple structural barriers to vaccination and timely healthcare [56,64]. Indigenous patients in the SAEH hospital discharge data experienced higher complication rates (50.3% vs. 41.6%; *p* = 0.033) and longer hospitalizations, reinforcing surveillance-based findings across an independent data source.

Vaccine effectiveness of 98.1% (95% CI: 98.0–98.2%) is consistent with expected values for measles-containing vaccines [3] and with estimates from recent outbreaks in the United States [58]. The population attributable fraction of 97.8%—indicating that an estimated 6741 of 6892 cases were attributable to lack of vaccination—underscores that this was an outbreak of access, not of vaccine failure. The absence of correlation between state-level vaccination coverage and incidence (rho = −0.13; *p* = 0.46) reflects the focal nature of transmission in “pockets of susceptibles”, where 81.2% of cases in municipalities with ≥10 cases occurred in settings where ≥80% of cases were unvaccinated (Figure 5). This pattern has been documented in US outbreaks where city-wide or state-wide coverage exceeded 90%, but sub-county pockets of undervaccination sustained transmission [11,24,25]. Chihuahua’s chronically low coverage (mean, 77.6%; only 65.6% in 2023; 15 years below 80%) illustrates how sustained coverage deficits create conditions for explosive transmission once the virus is introduced [1].

Wave segmentation—separating the January 2026 resurgence from the 2025 epidemic—revealed that late outbreak phase was independently associated with complications (aOR 1.68, 95% CI: 1.42–2.00; *p* < 0.001), a finding obscured in models without wave stratification (previously aOR 1.09; *p* = 0.366). This suggests that as the epidemic migrated from periurban areas into remote communities, affected populations faced compounding disadvantages: younger unvaccinated cohorts, limited healthcare access, and delayed case detection. The resurgence wave was not independently associated with increased complications (aOR 0.81; *p* = 0.164), and its lower complication rate (8.5% vs. 16.4% in Wave 1) likely reflects shifts in the geographic and demographic composition of cases—predominantly affecting Jalisco, Chiapas, and Sinaloa—rather than a secular trend in virulence. The marked differences in effective reproduction number between Chihuahua (peak Rt, 13.0) and other states also reflect differences in the size and density of susceptible populations: decades of coverage deficits in communities created large susceptible pools sustaining prolonged transmission, whereas later-affected states experienced smaller, more contained chains seeded by imported cases, with Rt declining more rapidly.

This study has several limitations that should be acknowledged. First, vaccination status was self-reported by patients or caregivers and may be subject to recall bias; misclassification could lead to underestimation of vaccine effectiveness if vaccinated cases were incorrectly classified as unvaccinated, and municipal averages may mask important intra-municipal heterogeneity. Second, the screening method for vaccine effectiveness estimation assumes that cases are representative of the population with respect to vaccination status, an assumption that may not hold in outbreak settings where vaccination campaigns modify coverage differentially across populations. Third, our ecological analyses linking individual cases to municipal-level social determinants may be subject to ecological fallacy; municipal averages may mask important intra-municipal heterogeneity, particularly in large or diverse municipalities. Fourth, the cross-sectional design limits causal inference regarding social determinants and transmission; we cannot definitively establish whether marginalization or school non-attendance caused increased transmission, or whether common underlying factors explain both. While statistically supported, this requires prospective confirmation. Fifth, genomic data covered only 207 sequences (~3% of cases); although geographic representation was adequate across 25 states, limited public data availability precluded phylogenetic analysis of intra-state transmission chains. Sixth, the SAEH hospital discharge data cover only Ministry of Health facilities through December 2025, excluding private-sector and social-security hospitals as well as the January 2026 resurgence, and therefore underestimates total hospitalization burden. Finally, complication rates may be underestimated, as the surveillance system captures acute complications but may miss delayed sequelae.

Despite these limitations, this study integrates individual-level surveillance data with municipal-level social determinants from eight national databases, enabling analysis of structural factors at a granularity rarely achieved in outbreak investigations. The dual-scale spatial analysis, wave-stratified LISA clustering, and dual logistic regression models provide methodological approaches transferable to other post-elimination settings. The identification of school non-attendance as a mediator of the marginalization–susceptibility pathway and the documentation of a three-stage transmission model generate evidence with direct policy relevance for outbreak response in Latin America and globally.

Several research directions emerge from our findings. First, prospective seroprevalence studies are needed to accurately quantify population immunity at subnational levels, particularly in communities identified as “pockets of susceptibles” to guide targeted vaccination campaigns. Second, qualitative research exploring vaccine decision-making in Mennonite, indigenous, and agricultural worker communities would inform culturally appropriate interventions. Third, mathematical modeling studies could estimate the vaccination coverage required to prevent future outbreaks in the specific demographic and spatial context of northern Mexico, accounting for population mobility and community structure. Fourth, implementation research should evaluate strategies for reaching mobile populations, including seasonal agricultural workers, with vaccination services. Fifth, enhanced molecular surveillance with real-time whole-genome sequencing could enable more precise tracking of transmission chains and earlier identification of new importations. Sixth, economic analyses quantifying the direct and indirect costs of this outbreak would strengthen the case for investment in elimination maintenance. Finally, cross-border surveillance coordination mechanisms between Mexico, the United States, and Canada warrant systematic evaluation to develop sustainable approaches for the post-elimination era.

## 5. Conclusions

Mexico’s 2025–2026 measles epidemic—6892 confirmed cases across 266 municipalities in all 32 states—represents the largest resurgence since interruption of endemic transmission and underscores that national coverage averages can mask focal vulnerability. Transmission was driven by accumulation of susceptible individuals in municipal pockets rather than by vaccine failure (VE, 98.1%). Molecular evidence confirmed at least two independent introductions dominated by a D8 lineage shared with the 2024–2025 North American outbreak, highlighting the role of cross-border mobility networks. The epidemic trajectory was socially patterned: introduction compatible with agricultural worker networks, amplification in undervaccinated communities, and progressive concentration among indigenous populations where the burden of complications was disproportionate. A key finding was that school non-attendance among children mediated the effect of very high marginalization on municipal case presence, identifying children not reached by school-based vaccination as a critical target population. Wave segmentation revealed that late outbreak phase was an independent risk factor for complications (aOR, 1.68), highlighting the compounding disadvantages faced by remote communities reached last by the epidemic. Sustaining elimination in Mexico requires shifting from coverage averages to precision public health: identifying municipal susceptibility pockets through linked sociodemographic surveillance, deploying catch-up vaccination targeting school non-attenders and mobile agricultural populations, and strengthening cross-border genomic surveillance in the post-elimination era.

## Figures and Tables

**Table 1:** Demographic and clinical characteristics of confirmed measles cases by geographic concentration, in Mexico, for epidemiological weeks August 2025–March 2026.

Characteristic	Chihuahua n = 4497 ^1^	Other States n = 2395 ^1^	Total n = 6892 ^1^	*p*-Value ^2^
Age, years	20 (4–31)	12 (4–24)	17 (4–29)	<0.001
Age group				<0.001
<1 year	490 (10.9%)	200 (8.4%)	690 (10.0%)	
1–4 years	643 (14.3%)	408 (17.0%)	1051 (15.2%)	
5–9 years	381 (8.5%)	416 (17.4%)	797 (11.6%)	
10–19 years	685 (15.2%)	567 (23.7%)	1252 (18.2%)	
20–39 years	1866 (41.5%)	666 (27.8%)	2532 (36.7%)	
≥40 years	432 (9.6%)	138 (5.8%)	570 (8.3%)	
Sex				0.022
Female	2161 (48.1%)	1221 (51.0%)	3382 (49.1%)	
Male	2336 (51.9%)	1174 (49.0%)	3510 (50.9%)	
Vaccination status				<0.001
Unvaccinated	3911 (87.0%)	1984 (82.8%)	5895 (85.5%)	
Vaccinated	586 (13.0%)	411 (17.2%)	997 (14.5%)	
Indigenous status	1247 (27.7%)	756 (31.6%)	2003 (29.1%)	<0.001
Case origin				<0.001
Import related	3515 (78.2%)	478 (20.0%)	3993 (57.9%)	
Imported	9 (0.2%)	246 (10.3%)	255 (3.7%)	
Unknown source	973 (21.6%)	1671 (69.8%)	2644 (38.4%)	
Complications	860 (19.1%)	209 (8.7%)	1069 (15.5%)	<0.001
Death	23 (0.5%)	2 (0.1%)	25 (0.4%)	0.009

^1^ Median (Q1–Q3); n (%). ^2^ Wilcoxon rank sum test; Pearson’s Chi-squared test.

**Table 2:** Municipal-level social determinants in municipalities with and without confirmed measles cases: Mexico, 2025.

	Municipality Status			
Variable	Overall n = 2469 ^1^	Without Cases n = 2203 ^1^	With Cases n = 266 ^1^	*p*-Value ^2^
Population (2025 projection)	13,981.0 (4702.0–36,621.0)	12,253.0 (4228.0–31,010.0)	43,463.5 (20,021.0–162,318.0)	<0.001
Marginalization index (0–100)	0.9 (0.8–0.9)	0.8 (0.8–0.9)	0.9 (0.8–0.9)	<0.001
Marginalization degree				<0.001
Very low	655 (26.5)	521 (23.6)	134 (50.4)	
Low	530 (21.5)	486 (22.1)	44 (16.5)	
Medium	494 (20.0)	467 (21.2)	27 (10.2)	
High	586 (23.7)	561 (25.5)	25 (9.4)	
Very high	204 (8.3)	168 (7.6)	36 (13.5)	
Illiteracy rate	8.2 (4.4–13.8)	8.4 (4.7–13.9)	5.4 (2.7–11.2)	<0.001
No basic education	46.3 (35.7–55.9)	46.9 (36.4–55.9)	41.6 (27.3– 54.9)	<0.001
Income < 2 min wages	84.6 (74.6–91.6)	85.5 (76.5–91.9)	74.1 (63.8–84.8)	<0.001
No drainage	1.4 (0.7–3.3)	1.5 (0.7–3.4)	0.9 (0.3–2.5)	<0.001
No electricity	0.8 (0.4–1.7)	0.9 (0.4–1.7)	0.5 (0.2–1.3)	<0.001
No piped water	2.5 (0.9–7.3)	2.6 (0.9–7.4)	1.6 (0.6–6.3)	<0.001
Dirt floor	4.7 (1.7–11.0)	4.9 (1.8–11.2)	2.4 (1.0–8.3)	<0.001
No health insurance	22.6 (16.2–30.6)	22.7 (16.2–30.7)	22.5 (16.3–29.8)	0.929
Social lag index	−0.2 (−0.8–0.5)	−0.2 (−0.7–0.5)	−0.7 (−1.1–0.0)	<0.001
Social lag degree				<0.001
Very low	677 (27.4)	546 (24.8)	131 (49.2)	
Low	893 (36.2)	822 (37.3)	71 (26.7)	
Medium	504 (20.4)	484 (22.0)	20 (7.5)	
High	243 (9.8)	229 (10.4)	14 (5.3)	
Very high	152 (6.2)	122 (5.5)	30 (11.3)	
Rural population	100.0 (40.1–100.0)	100.0 (43.4–100.0)	44.0 (14.9–77.9)	<0.001
Overcrowding	25.0 (18.7–32.8)	25.4 (19.3–32.9)	20.5 (14.5–30.9)	<0.001
Households with remittances	5.8 (2.6–12.9)	5.9 (2.6–13.5)	5.1 (2.0–10.5)	0.009
Migration intensity index	63.9 (62.2–64.7)	63.8 (62.1–64.7)	64.0 (62.8–64.8)	0.026
Migration intensity degree				0.002
None	12 (0.5)	12 (0.5)	0 (0.0)	
Very low	861 (34.9)	764 (34.7)	97 (36.5)	
Low	686 (27.8)	599 (27.2)	87 (32.7)	
Medium	432 (17.5)	379 (17.2)	53 (19.9)	
High	341 (13.8)	315 (14.3)	26 (9.8)	
Very high	137 (5.5)	134 (6.1)	3 (1.1)	
Agricultural units with day laborers	47.7 (36.4–59.3)	48.0 (36.9–60.0)	42.8 (33.1–54.2)	<0.001
Day laborers tertile				<0.001
T1 (Low)	818 (33.3)	709 (32.3)	109 (42.6)	
T2 (Medium)	818 (33.3)	731 (33.3)	87 (34.0)	
T3 (High)	818 (33.3)	758 (34.5)	60 (23.4)	

^1^ n (%); median (Q1–Q3). ^2^ Wilcoxon rank sum test; Pearson’s Chi-squared test.

**Table 3:** Case characteristics and municipal determinants by outbreak phase (relative weeks): Mexico 2025, measles outbreak.

Outbreak Phase (Relative Weeks per State)								
Variable	Overall n = 6892 ^1^	Introduction (Rel Wk 1–4) n = 274 ^1^	Growth (Rel Wk 5–11) n = 1621 ^1^	Peak (Rel Wk 12–14) n = 981 ^1^	Decline (Rel Wk 15–24) n = 2195 ^1^	Late (Rel Wk 25+) n = 1080 ^1^	Resurgence (Wave 2) n = 741 ^1^	*p*-Value ^2^
Age (years)	17.0 (4.0–29.0)	10.0 (3.0–21.0)	22.0 (8.0–31.0)	22.0 (7.0–32.0)	15.0 (3.0–28.0)	11.0 (2.0–23.0)	15.0 (6.0–28.0)	<0.001
Sex								0.182
Female	3510 (51%)	132 (48%)	842 (52%)	528 (54%)	1116 (51%)	528 (49%)	364 (49%)	
Male	3382 (49%)	142 (52%)	779 (48%)	453 (46%)	1079 (49%)	552 (51%)	377 (51%)	
Unvaccinated	5895 (86%)	234 (85%)	1393 (86%)	810 (83%)	1918 (87%)	928 (86%)	612 (83%)	0.002
Indigenous	2003 (29%)	49 (18%)	94 (5.8%)	129 (13%)	902 (41%)	711 (66%)	118 (16%)	<0.001
Complications	1069 (16%)	28 (10%)	146 (9.0%)	96 (9.8%)	486 (22%)	250 (23%)	63 (8.5%)	<0.001
Death	25 (0.4%)	0 (0%)	5 (0.3%)	1 (0.1%)	10 (0.5%)	9 (0.8%)	0 (0%)	0.025
Marginalization index (0–100) *	0.9 (0.9–0.9)	0.9 (0.9–0.9)	0.9 (0.9–0.9)	0.9 (0.9–0.9)	0.9 (0.9–0.9)	0.9 (0.9–0.9)	0.9 (0.9–0.9)	<0.001
Marginalization degree *								<0.001
Very low	5138 (75%)	218 (80%)	1505 (93%)	859 (88%)	1398 (64%)	646 (60%)	512 (69%)	
Low	750 (11%)	33 (12%)	83 (5.1%)	46 (4.7%)	311 (14%)	131 (12%)	146 (20%)	
Medium	124 (1.8%)	13 (4.8%)	7 (0.4%)	3 (0.3%)	17 (0.8%)	65 (6.0%)	19 (2.6%)	
High	211 (3.1%)	3 (1.1%)	11 (0.7%)	25 (2.5%)	98 (4.5%)	47 (4.4%)	27 (3.6%)	
Very high	666 (9.7%)	4 (1.5%)	15 (0.9%)	48 (4.9%)	371 (17%)	191 (18%)	37 (5.0%)	
Illiteracy rate (%) *	2.1 (1.8–5.8)	3.0 (1.8, 6.4)	1.8 (1.8–3.0)	1.8 (1.7–2.6)	2.6 (1.8–9.1)	3.0 (1.9–9.7)	2.3 (1.9–7.9)	<0.001
No basic education (%) *	35.3 (24.3–46.2)	37.5 (32.3–45.6)	35.3 (28.5–39.5)	35.3 (24.0–39.5)	35.3 (26.9–55.1)	35.8 (24.2–58.0)	26.9 (23.4–38.4)	<0.001
Income < 2 min wages (%) *	62.9 (51.3–77.0)	66.0 (55.0–76.1)	51.3 (51.3–66.0)	51.3 (51.3–69.3)	68.9 (51.3–81.6)	70.3 (59.8–81.9)	59.1 (49.6–74.6)	<0.001
No drainage (%) *	0.2 (0.1–0.7)	0.6 (0.2–0.9)	0.2 (0.2–0.4)	0.2 (0.1–0.3)	0.2 (0.1–1.7)	0.5 (0.2–3.3)	0.1 (0.0–0.5)	<0.001
No electricity (%) *	0.2 (0.1–0.4)	0.3 (0.2–0.4)	0.1 (0.1–0.3)	0.1 (0.1–0.2)	0.2 (0.1–1.0)	0.3 (0.1–2.0)	0.1 (0.1–0.4)	<0.001
No piped water (%) *	0.6 (0.4–1.0)	0.9 (0.4–1.3)	0.4 (0.4–0.9)	0.5 (0.4–0.9)	0.6 (0.4–3.4)	0.9 (0.3–3.4)	0.6 (0.5–4.9)	<0.001
Dirt floor (%) *	0.6 (0.3–2.3)	0.7 (0.6–1.9)	0.5 (0.3–0.6)	0.5 (0.3–1.3)	0.6 (0.4–5.1)	0.7 (0.4–7.0)	2.3 (1.5–6.3)	<0.001
No health insurance (%) *	15.4 (13.1–23.0)	19.8 (13.1–39.9)	13.1 (13.1–17.8)	13.1 (13.1–19.0)	15.4 (13.1–19.7)	14.1 (10.9–18.3)	29.7 (27.5–35.4)	<0.001
Social lag index *	−1.1 (−1.2–−0.6)	−0.9 (−1.1–−0.6)	−1.1 (−1.2–−0.9)	−1.1 (−1.2–−1.0)	−1.1 (−1.2–−0.2)	−1.0 (−1.3–−0.2)	−1.2 (−1.2–−0.5)	<0.001
Social lag degree *								<0.001
Very low	4982 (72%)	202 (75%)	1442 (89%)	839 (86%)	1373 (63%)	628 (58%)	498 (67%)	
Low	1022 (15%)	62 (23%)	151 (9.3%)	69 (7.0%)	351 (16%)	213 (20%)	176 (24%)	
Medium	193 (2.8%)	3 (1.1%)	13 (0.8%)	25 (2.5%)	84 (3.8%)	48 (4.4%)	20 (2.7%)	
High	53 (0.8%)	0 (0%)	0 (0%)	0 (0%)	38 (1.7%)	0 (0%)	15 (2.0%)	
Very high	639 (9.3%)	4 (1.5%)	15 (0.9%)	48 (4.9%)	349 (16%)	191 (18%)	32 (4.3%)	
Rural population < 5000 (%) *	19.3 (5.6–38.0)	26.0 (19.3–49.7)	19.3 (7.0–20.2)	19.3 (3.3–19.3)	19.3 (5.6–54.2)	19.1 (10.3–63.8)	6.4 (1.6–18.9)	<0.001
Migration intensity index *	63.7 (63.4–64.2)	63.9 (63.3–64.3)	63.4 (63.4–63.7)	63.4 (63.4–63.7)	63.7 (63.4–64.3)	63.7 (63.2–64.1)	64.4 (64.1–64.8)	<0.001
Migration intensity degree *								<0.001
None	0 (0%)	0 (0%)	0 (0%)	0 (0%)	0 (0%)	0 (0%)	0 (0%)	
Very low	1193 (17%)	53 (20%)	91 (5.6%)	75 (7.6%)	461 (21%)	141 (13%)	372 (50%)	
Low	4545 (66%)	151 (56%)	1306 (81%)	707 (72%)	1397 (64%)	693 (64%)	291 (39%)	
Medium	993 (14%)	42 (15%)	189 (12%)	165 (17%)	312 (14%)	235 (22%)	50 (6.7%)	
High	151 (2.2%)	23 (8.5%)	33 (2.0%)	34 (3.5%)	24 (1.1%)	11 (1.0%)	26 (3.5%)	
Very high	7 (0.1%)	2 (0.7%)	2 (0.1%)	0 (0%)	1 (<0.1%)	0 (0%)	2 (0.3%)	
Agricultural day laborers (%) *	32.2 (31.3–42.1)	45.8 (32.2–53.6)	32.2 (32.2–34.4)	32.2 (31.3–33.7)	32.2 (31.2–39.9)	33.3 (31.3–51.5)	48.4 (31.3–51.5)	<0.001
High day laborer municipality (>50%) *	1170 (17%)	109 (40%)	185 (12%)	68 (7.1%)	294 (14%)	323 (30%)	191 (29%)	<0.001

^1^ Median (Q1–Q3); mean (SD); n (%). * Municipal-level indicator from residence municipality. Relative week: Week 1 = first week with cases in each state. ^2^ Kruskal–Wallis rank sum test; Pearson’s Chi-squared test.

**Table 4:** Risk factors for measles complications: multivariable logistic regression: Mexico, 2025–2026 (n = 6892).

Variable	Complications n/N (%)	aOR (95% CI)	*p*-Value
Age group			
5–19 years (ref)	281/2048 (13.7%)	1.00 (ref)	-
<1 year	244/690 (35.4%)	3.36 (2.72–4.15)	<0.001
1–4 years	306/1051 (29.1%)	2.58 (2.14–3.13)	<0.001
≥20 years	238/3100 (7.7%)	0.64 (0.53–0.77)	<0.001
Indigenous			
No (ref)	521/4886 (10.7%)	1.00 (ref)	-
Yes	548/2003 (27.4%)	1.89 (1.61–2.22)	<0.001
Vaccination status			
Vaccinated (ref)	81/997 (8.1%)	1.00 (ref)	-
Unvaccinated	988/5892 (16.8%)	1.96 (1.53–2.51)	<0.001
Outbreak phase			
Early, weeks 1–14 (ref)	333/3614 (9.2%)	1.00 (ref)	-
Late, weeks ≥ 15	736/3275 (22.5%)	1.68 (1.42–2.00)	<0.001
Municipality type			
Urban (ref)	668/5429 (12.3%)	1.00 (ref)	-
Rural (>50% in localities < 5000)	401/1460 (27.5%)	1.73 (1.48–2.03)	<0.001
Epidemic wave			
Wave 1, 2025 (ref)	1006/6148 (16.4%)	1.00 (ref)	-
Resurgence, January 2026	63/741 (8.5%)	0.81 (0.60–1.09)	0.164

Model fit: Pseudo R^2^ = 0.136; AIC = 5154.9. aOR = adjusted odds ratio; CI = confidence interval. Rural municipality defined as >50% population in localities of <5000 inhabitants. Resurgence wave: cases from epidemiological weeks 54–56 (January 2026). Complications defined as pneumonia, otitis media, encephalitis, or other.

## Data Availability

The primary and secondary datasets analyzed in this study are publicly available from official Mexican government open data portals and international repositories. The R scripts used for data linkage, statistical analysis, and figure generation are available from the corresponding author upon request.

## Supplementary Materials

The following supporting information can be downloaded at: https://www.mdpi.com/article/10.3390/v18020219/s1, Supplementary Figures: Supplementary Figure S1. State-level LISA cluster analysis of measles incidence at the municipal level, Mexico, 2025–2026; Supplementary Figure S2. Vaccination coverage and vaccine effectiveness during the measles outbreak, Mexico, 2025–2026; Supplementary Figure S3. Measles virus genotype distribution in Mexico: genomic surveillance data from GenBank (2011–2023) and SSA epidemiological bulletins (2025). Supplementary Tables: Supplementary Table S1. Data dictionary of variables from linked national open-access databases used in the analysis; Supplementary Table S2. Demographic and clinical characteristics of confirmed measles cases by age group: measles outbreak, Mexico, 2025–2026; Supplementary Table S3. Demographic and clinical characteristics of confirmed measles cases by complication status: measles outbreak, Mexico, 2025–2026; Supplementary Table S4. Demographic and clinical characteristics of confirmed measles cases by outbreak phase: measles outbreak, Mexico, 2025–2026; Supplementary Table S5. Summary of effective reproduction number (Rt) estimates by geographic region and extended methodology: measles outbreak, Mexico, 2025–2026; Supplementary Table S6. Spatial analysis of measles outbreak clustering and diffusion: Mexico, 2025–2026; Supplementary Table S7. Comparison of logistic regression models for municipal measles case presence (≥1 confirmed case): Mexico, 2025–2026; Supplementary Table S8. Social determinants of measles cases stratified by state: Mexico, 2025–2026; Supplementary Table S9. Sensitivity analysis comparing the first 50 and last 50 municipalities affected during the measles outbreak: Mexico, 2025–2026; Supplementary Table S10. National vaccination metrics and vaccine effectiveness for the measles outbreak: Mexico, 2025–2026; Supplementary Table S11. Vaccine effectiveness by state (states with ≥20 cases); Supplementary Table S12. Bivariable analysis of risk factors for measles complications: Mexico, 2025–2026; Supplementary Table S13. Hospital discharge characteristics of measles cases from Ministry of Health (SSA) facilities: Mexico, 2025 (n = 663). Supplementary Notes: Supplementary Note S1. Estimation of the effective reproduction number (Rt).

## Abbreviations

The following abbreviations are used in this manuscript:

aOR	Adjusted odds ratio
AIC	Akaike information criterion
CFR	Case fatality rate
CENSIA	Centro Nacional para la Salud de la Infancia y la Adolescencia (National Center for Child and Adolescent Health)
CI	Confidence interval
CONAPO	Consejo Nacional de Población (National Population Council)
CONEVAL	Consejo Nacional de Evaluación de la Pol√≠tica de Desarrollo Social (National Council for the Evaluation of Social Development Policy)
CrI	Credible Interval
DALYs	Disability-adjusted life years
DGE	Dirección General de Epidemiología (General Directorate of Epidemiology)
DGIS	Dirección General de Informacion en Salud (General Directorate of Health Information)
EFEs	Sistema Especial de Vigilancia Epidemiológica de Enfermedades Febriles Exantematicas (Special Surveillance System for Febrile Exanthematous Diseases)
ICD-10	International Classification of Diseases 10th Revision
IgM	Immunoglobulin M
INEGI	Instituto Nacional de Estadística y Geografía (National Institute of Statistics and Geography)
IQR	Interquartile range
IRR	Incidence rate ratio
LISA	Local indicators of spatial association
LOESS	Locally estimated scatterplot smoothing
MCV1	Measles-containing vaccine first dose
MMR	Measles-mumps-rubella vaccine
NCBI	National Center for Biotechnology Information
NOM	Norma Oficial Mexicana (Mexican Official Standard)
OR	Odds ratio
PAF	Population attributable fraction
PAHO	Pan American Health Organization
PCV	Proportion of cases vaccinated
PPV	Population proportion vaccinated
R0	Basic reproduction number
RR	Relative risk
RT-PCR	Reverse transcription polymerase chain reaction
Rt	Effective reproduction number
SAEH	Subsistema Automatizado de Egresos Hospitalarios (Automated Hospital Discharge Subsystem)
SD	Standard deviation
SE	Semana epidemiologica (epidemiological week)
SINAVE	Sistema Nacional de Vigilancia Epidemiológica (National Epidemiological Surveillance System)
VE	Vaccine effectiveness
VIF	Variance inflation factor
WHO	World Health Organization

## References

[1] Do L.A.H., Mulholland K. (2025). Measles 2025. N. Engl. J. Med..

[2] Stoneman E.K. (2025). Measles. JAMA.

[3] Hübschen J.M., Gouandjika-Vasilache I., Dina J. (2022). Measles. Lancet.

[4] Bester J.C. (2016). Measles and Measles Vaccination: A Review. JAMA Pediatr..

[5] Chen W., Du M., Deng J., Liu M., Liu J. (2025). Global, Regional, and National Trends of Measles Burden and Its Vaccination Coverage among Children under 5 Years Old: An Updated Systematic Analysis from the Global Burden of Disease Study 2021. Int. J. Infect. Dis..

[6] Peltola H. (2025). The History of Measles and Vaccine Development. Acta Paediatr..

[7] Laksono B.M., de Vries R.D., Verburgh R.J., Visser E.G., de Jong A., Fraaij P.L.A., Ruijs W.L.M., Nieuwenhuijse D.F., van den Ham H.-J., Koopmans M.P.G. (2018). Studies into the Mechanism of Measles-Associated Immune Suppression during a Measles Outbreak in the Netherlands. Nat. Commun..

[8] Mina M.J., Kula T., Leng Y., Li M., de Vries R.D., Knip M., Siljander H., Rewers M., Choy D.F., Wilson M.S. (2019). Measles Virus Infection Diminishes Preexisting Antibodies That Offer Protection from Other Pathogens. Science.

[9] Gastañaduy P.A., Goodson J.L., Panagiotakopoulos L., Rota P.A., Orenstein W.A., Patel M. (2021). Measles in the 21st Century: Progress Toward Achieving and Sustaining Elimination. J. Infect. Dis..

[10] Mattingly T.J. (2025). Penny Wise, Pound Foolish: The Cost of Reduced Support for Measles Prevention. Vaccine.

[11] (2021). Local Burden of Disease Vaccine Coverage Collaborators. Mapping Routine Measles Vaccination in Low- and Middle-Income Countries. Nature.

[12] Masresha B.G., Hatcher C., Lebo E., Tanifum P., Bwaka A.M., Minta A.A., Antoni S., Grant G.B., Perry R.T., O’Connor P. (2023). Progress Toward Measles Elimination—African Region, 2017–2021. MMWR. Morb. Mortal. Wkly. Rep..

[13] Guerra F.M., Bolotin S., Lim G., Heffernan J., Deeks S.L., Li Y., Crowcroft N.S. (2017). The Basic Reproduction Number (R0) of Measles: A Systematic Review. Lancet Infect. Dis..

[14] Jones C.E., Danovaro-Holliday M.C., Mwinnyaa G., Gacic-Dobo M., Francis L., Grevendonk J., Nedelec Y., Wallace A., Sodha S.V., Sugerman C. (2024). Routine Vaccination Coverage—Worldwide, 2023. MMWR. Morb. Mortal. Wkly. Rep..

[15] Marziano V., Bella A., Menegale F., Del Manso M., Petrone D., Palamara A.T., Pezzotti P., Merler S., Filia A., Poletti P. (2025). Estimating Measles Susceptibility and Transmission Patterns in Italy: An Epidemiological Assessment. Lancet Infect. Dis..

[16] Filia A., Del Manso M., Petrone D., Magurano F., Gioacchini S., Pezzotti P., Palamara A.T., Bella A. (2025). Surge in Measles Cases in Italy from August 2023 to January 2025: Characteristics of Cases and Public Health Relevance. Vaccines.

[17] González-Parra G., Vestrand A., Mujynya R. (2025). Modeling and Characterizing the Growth of the Texas–New Mexico Measles Outbreak of 2025. Epidemiologia.

[18] Rey-Benito G., Pastor D., Whittembury A., Durón R., Pacis-Tirso C., Bravo-Alcántara P., Ortiz C., Andrus J. (2024). Sustaining the Elimination of Measles, Rubella and Congenital Rubella Syndrome in the Americas, 2019-2023: From Challenges to Opportunities. Vaccines.

[19] Carnalla M., Gaspar-Castillo C., Dimas-González J., Aparicio-Antonio R., Justo-Berrueta P.S., López-Martínez I., Shamah-Levy T., Lazcano-Ponce E., Barrientos-Gutiérrez T., Alpuche-Aranda C.M. (2025). A Population-Based Measles Serosurvey in Mexico: Implications for Re-Emergence. Vaccine.

[20] Hersh B.S., Tambini G., Nogueira A.C., Carrasco P., de Quadros C.A. (2000). Review of Regional Measles Surveillance Data in the Americas, 1996–1999. Lancet.

[21] Santos J.I., Nakamura M.A., Godoy M.V., Kuri P., Lucas C.A., Conyer R.T. (2004). Measles in Mexico, 1941–2001: Interruption of Endemic Transmission and Lessons Learned. J. Infect. Dis..

[22] Rivadeneira M.F., Bassanesi S.L., Fuchs S.C. (2018). Socioeconomic Inequalities and Measles Immunization Coverage in Ecuador: A Spatial Analysis. Vaccine.

[23] Fene F., Johri M., Michel M.E., Reyes-Morales H., Pelcastre-Villafuerte B.E. (2025). Multiple Deprivations as Drivers of Suboptimal Basic Child Vaccination in Latin America and the Caribbean: Cross-Sectional Analysis of Household Survey Data for 18,136 Children across 211 Regions in 15 Countries. Int. J. Equity Health.

[24] Masters N.B., Eisenberg M.C., Delamater P.L., Kay M., Boulton M.L., Zelner J. (2020). Fine-Scale Spatial Clustering of Measles Nonvaccination That Increases Outbreak Potential Is Obscured by Aggregated Reporting Data. Proc. Natl. Acad. Sci. USA.

[25] Robert A., Kucharski A.J., Funk S. (2022). The Impact of Local Vaccine Coverage and Recent Incidence on Measles Transmission in France between 2009 and 2018. BMC Med..

[26] Secretaria de Salud (Mexico), Direccion General de Epidemiologia Datos Abiertos: Bases Historicas de Enfermedades Febriles Exantematicas. Gobierno de Mexico. https://www.gob.mx/salud/documentos/datos-abiertos-bases-historicas-de-enfermedades-febriles-exantematicas.

[27] Diario Oficial de la Federacion (DOF) (2013). Norma Oficial Mexicana NOM-017-SSA2-2012, para la Vigilancia Epidemiologica.

[28] Instituto de Diagnostico y Referencia Epidemiologicos (InDRE), Red Nacional de Laboratorios de Salud Publica (RNLSP) Lineamientos para la Vigilancia por Laboratorio de la Enfermedad Febril Exantematica (EFE) (LVL_EFE_4T). Version 1. Mexico. https://www.gob.mx/cms/uploads/attachment/file/487585/LVL_EFE_4T.pdf.

[29] Secretaria de Salud (Mexico), Direccion General de Informacion en Salud (DGIS) Egresos Hospitalarios: Cubos Dinamicos (SAEH). http://dgis.salud.gob.mx/contenidos/basesdedatos/bdc_egresoshosp_gobmx.html.

[30] Índices de Marginación—Base de Datos—Datos.Gob.Mx. https://datos.gob.mx/dataset/indices_marginacion.

[31] Índice Rezago Social 2020. https://www.coneval.org.mx/Medicion/IRS/Paginas/Indice_Rezago_Social_2020.aspx.

[32] Consejo Nacional de Poblacion (CONAPO) Indices de Intensidad Migratoria Mexico-Estados Unidos 2020. Gobierno de Mexico. https://www.gob.mx/conapo/documentos/indices-de-intensidad-migratoria-mexico-estados-unidos-2020.

[33] Geografía(INEGI), I.N. de E. y Censo Agropecuario (CA) 2022. https://www.inegi.org.mx/programas/ca/2022/#datos_abiertos.

[34] Catálogo Nacional de Indicadores. https://www.snieg.mx/cni/escenario.aspx?idOrden=1.4&ind=6300000008&gen=143&d=n.

[35] GenBank Overview. https://www.ncbi.nlm.nih.gov/genbank/.

[36] Secretaria de Salud (Mexico), Direccion General de Epidemiologia (DGE) Boletin Epidemiologico: Sistema Nacional de Vigilancia Epidemiologica (SINAVE)/Sistema Unico de Informacion (SUAVE). https://www.gob.mx/salud/documentos/boletinepidemiologico-sistema-nacional-de-vigilancia-epidemiologica-sistema-unico-de-informacion.

[37] Secretaria General del Consejo Nacional de Poblacion (CONAPO) Proyecciones de Poblacion: Indicadores Demograficos Especiales para los Municipios de Mexico, Desde 1990 a 2040 (Dataset). Datos.gob.mx (Gobierno de Mexico Open Data Portal). https://www.datos.gob.mx/dataset/proyecciones-de-poblacion/resource/99b28bb6-8e31-48e1-b162-85a7e4deafc3.

[38] Lessler J., Reich N.G., Brookmeyer R., Perl T.M., Nelson K.E., Cummings D.A.T. (2009). Incubation Periods of Acute Respiratory Viral Infections: A Systematic Review. Lancet Infect. Dis..

[39] Cori A., Ferguson N.M., Fraser C., Cauchemez S. (2013). A New Framework and Software to Estimate Time-Varying Reproduction Numbers During Epidemics. Am. J. Epidemiol..

[40] Thompson R.N., Stockwin J.E., van Gaalen R.D., Polonsky J.A., Kamvar Z.N., Demarsh P.A., Dahlqwist E., Li S., Miguel E., Jombart T. (2019). Improved Inference of Time-Varying Reproduction Numbers during Infectious Disease Outbreaks. Epidemics.

[41] Moran P.A.P. (1950). Notes on Continuous Stochastic Phenomena. Biometrika.

[42] Anselin L. (1995). Local Indicators of Spatial Association—LISA. Geogr. Anal..

[43] Pebesma E. (2018). Simple Features for R: Standardized Support for Spatial Vector Data. R J..

[44] Bivand R.S., Wong D.W.S. (2018). Comparing Implementations of Global and Local Indicators of Spatial Association. TEST.

[45] Farrington C.P. (1993). Estimation of Vaccine Effectiveness Using the Screening Method. Int. J. Epidemiol..

[46] Orenstein W.A., Bernier R.H., Dondero T.J., Hinman A.R., Marks J.S., Bart K.J., Sirotkin B. (1985). Field Evaluation of Vaccine Efficacy. Bull. World Health Organ..

[47] Orenstein W.A., Bernier R.H., Hinman A.R. (1988). Assessing Vaccine Efficacy in the Field: Further Observations. Epidemiol. Rev..

[48] Zucker J.R., Rosen J.B., Iwamoto M., Arciuolo R.J., Langdon-Embry M., Vora N.M., Rakeman J.L., Isaac B.M., Jean A., Asfaw M. (2020). Consequences of Undervaccination—Measles Outbreak, New York City, 2018–2019. N. Engl. J. Med..

[49] Manisha Patel M.D., Adria D., Lee M., Nakia S.C., Lemmons M.P.H., Redd S.B., Poser S., Debra Blog M.D., Jane R., Zucker M.D. (2019). National Update on Measles Cases and Outbreaks—United States, January 1–October 1, 2019. MMWR. Morb. Mortal. Wkly. Rep..

[50] Hall V. (2017). Measles Outbreak—Minnesota April–May 2017. MMWR. Morb. Mortal. Wkly. Rep..

[51] Prevention of Measles, Rubella, Congenital Rubella Syndrome, and Mumps, 2013. https://www.cdc.gov/mmwr/preview/mmwrhtml/rr6204a1.htm.

[52] Bursac Z., Gauss C.H., Williams D.K., Hosmer D.W. (2008). Purposeful Selection of Variables in Logistic Regression. Source Code Biol. Med..

[53] Hosmer D.W. (2013). Model-Building Strategies and Methods for Logistic Regression. Applied Logistic Regression.

[54] Measles—Region of the Americas. https://www.who.int/emergencies/disease-outbreak-news/item/2025-DON565.

[55] Pasadyn F., Mamo N., Caplan A. (2025). Battling Measles: Shifting Strategies to Meet Emerging Challenges and Inequities. Ethics Med. Public Health.

[56] Mathis A.D. (2025). Measles Update—United States, January 1–April 17, 2025. MMWR. Morb. Mortal. Wkly..

[57] Hewitt G.-L., Obeid A., Fischer P.R. (2025). Measles Outbreaks in the United States in 2025: Practice, Policy, and the Canary in the Coalmine. New Microbes New Infect..

[58] CDC Measles Cases and Outbreaks. https://www.cdc.gov/measles/data-research/index.html.

[59] Measles Outbreak—August 12, 2025|Texas DSHS. https://www.dshs.texas.gov/news-alerts/measles-outbreak-2025?utm_source=chatgpt.com.

[60] Pan American Health Organization (PAHO)/World Health Organization (WHO) (2025). PAHO Calls for Regional Action as the Americas Lose Measles Elimination Status. https://www.paho.org/en/news/10-11-2025-paho-calls-regional-action-americas-lose-measles-elimination-status.

[61] Pan American Health Organization (PAHO)/World Health Organization (WHO) (2026). Measles Elimination Status in the United States and Mexico. https://www.paho.org/en/news/16-1-2026-measles-elimination-status-united-states-and-mexico.

[62] Grenfell B.T., Bjørnstad O.N., Kappey J. (2001). Travelling Waves and Spatial Hierarchies in Measles Epidemics. Nature.

[63] Pan American Health Organization (PAHO)/World Health Organization (WHO) (2025). Ten Countries in the Americas Report Measles Outbreaks in 2025. https://www.paho.org/en/news/15-8-2025-ten-countries-americas-report-measles-outbreaks-2025.

[64] Lanke R., Chimurkar V. (2024). Measles Outbreak in Socioeconomically Diverse Sections: A Review. Cureus.

