# Supplementary material for: Social Determinants and Outbreak Dynamics of the 2025 Measles Epidemic in Mexico: A Nationwide Analysis of Linked Surveillance Data
Source: Viruses. 2026 Feb 8;18(2):219. doi: 10.3390/v18020219 (PMC12944898; doi:10.3390/v18020219)
Supplement: Supplementary file 1 [file viruses-18-00219-s001.zip › viruses-4120266-supplementary.pdf]

Supplementary Material

Supplementary Figures

Supplementary Figure S1. State-level LISA cluster analysis of measles incidence at the municipal level, Mexico, 2025–2026
Supplementary Figure S2. Vaccination coverage and vaccine effectiveness during the measles outbreak, Mexico 2025–2026.
Supplementary Figure S3. Measles Virus Genotype Distribution in Mexico, Genomic surveillance data from GenBank (2011–2023) and SSA Epidemiological Bulletins (2025)

Supplementary Tables

Supplementary Table S1. Data dictionary of variables from linked national open-access databases used in the analysis.
Supplementary Table S2. Demographic and clinical characteristics of confirmed measles cases by age group, measles outbreak, Mexico, 2025-2026.
Supplementary Table S3. Demographic and clinical characteristics of confirmed measles cases by complication status, measles outbreak, Mexico, 2025-2026.
Supplementary Table S4. Demographic and clinical characteristics of confirmed measles cases by outbreak phase, measles outbreak, Mexico, 2025-2026.
Supplementary Table S5. Summary of effective reproduction number (Rt) estimates by geographic region and extended methodology, measles outbreak, Mexico, 2025-2026.
Supplementary Table S6. Spatial analysis of measles outbreak clustering and diffusion, Mexico 2025–2026.
Supplementary Table S7. Comparison of logistic regression models for municipal measles case presence (≥1 confirmed case), Mexico 2025–2026.
Supplementary Table S8. Social determinants of measles cases stratified by state, Mexico 2025–2026.
Supplementary Table S9. Sensitivity analysis comparing the first 50 and last 50 municipalities affected during the measles outbreak, Mexico 2025–2026.
Supplementary Table S10. National vaccination metrics and vaccine effectiveness for the measles outbreak, Mexico 2025–2026.
Supplementary Table S11. Vaccine Effectiveness by state (states with >=20 cases)
Supplementary Table S12. Bivariable analysis of risk factors for measles complications, Mexico 2025-2026.
Supplementary Table S13. Hospital discharge characteristics of measles cases from Ministry of Health (SSA) facilities, Mexico 2025 (N = 663).

Supplementary Notes

Supplementary Note S1. Estimation of the Effective Reproduction Number (Rt)

Supplementary Figure S1. State-level LISA cluster analysis of measles incidence at the municipal level, Mexico, 2025–2026. Local Moran's I was computed within each state using queen contiguity weights (p < 0.05). Only states with ≥100 confirmed cases and ≥20 municipalities are shown.

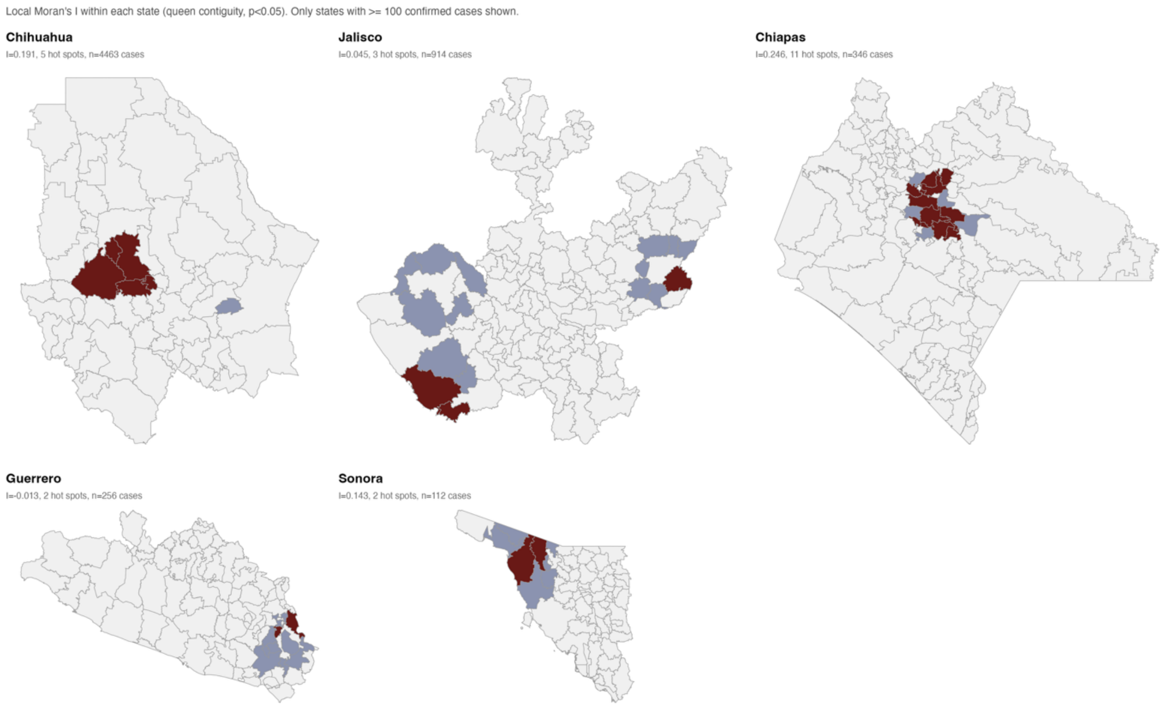

Spatial autocorrelation measures the degree to which observations at nearby geographic locations tend to be similar (positive autocorrelation) or dissimilar (negative autocorrelation). We assessed spatial clustering of measles incidence at the municipal level using both global and local indicators of spatial association.

The analysis was conducted at three levels:

1. **National (full period):** All 2,457 municipalities, full outbreak period (EW 8–56)
2. **Wave-stratified:** Wave 1 (EW 8–53/2025) vs Wave 2 (EW 1–3/2026), to assess changes in spatial clustering between epidemic phases
3. **State-level:** Within each state with ≥100 confirmed cases and ≥20 municipalities, to detect intra-state clustering patterns not visible at the national scale

The spatial unit of analysis was the municipality (*municipio*), the second-level administrative division in Mexico (N = 2,457 municipalities with valid geometry). Cumulative incidence rates were calculated as:

$$\text{Incidence rate}_i = (\text{Cases}_i \ / \ \text{Population}_i) \times 100{,}000$$

where Population_i corresponds to CONAPO 2025 mid-year population projections for each municipality. Municipalities with zero population or missing geometry were excluded.

Global Moran's I

Definition

Global Moran's I (Moran, 1950) summarizes the overall spatial autocorrelation across the study area. It is defined as:

$$I = (N \ / \ S_0) \times [\Sigma_i \Sigma_j \, w_{ij}(x_i - \bar{x})(x_j - \bar{x})] \ / \ [\Sigma_i (x_i - \bar{x})^2]$$

where:

- N is the number of spatial units (municipalities)
- x_i is the incidence rate at municipality i
- $\bar{x}$ is the mean incidence rate across all municipalities
- w_{ij} is the spatial weight between municipalities i and j

- $S_0 = \Sigma_i \Sigma_j \, w_{ij}$ is the sum of all spatial weights

Interpretation

Value	Interpretation
$I > 0$	Positive spatial autocorrelation: similar values cluster together
$I \approx 0$	Random spatial pattern (no autocorrelation)
$I < 0$	Negative spatial autocorrelation: dissimilar values cluster together
Expected value	$E[I] = -1/(N-1)$, approximately 0 for large N

Inference

Statistical significance was assessed using the analytical normal approximation implemented in moran.test():

$$z(I) = (I - E[I]) \ / \ SD[I]$$

where $E[I]$ and $SD[I]$ are the expected value and standard deviation under the null hypothesis of spatial randomness (complete spatial randomness, CSR). The p-value corresponds to a one-sided test (alternative: greater), testing for positive spatial autocorrelation

Local Indicators of Spatial Association (LISA)

Definition

The Local Moran's *I* statistic (Anselin, 1995) decomposes the global Moran's *I* into contributions from each spatial unit, identifying local clusters and spatial outliers:

$$I_i = [(x_i - \bar{x}) \ / \ s^2] \times \Sigma_j \, w_{ij}(x_j - \bar{x})$$

where $s^2 = (1/N)\, \Sigma_i(x_i - \bar{x})^2$ is the variance of the variable.

The key property of LISA statistics is that the sum of all local indicators is proportional to the global indicator:

$$\Sigma_i \, I_i = \text{constant} \times I$$

Cluster Classification

Each municipality was classified based on the combination of its own value relative to the mean and the spatially lagged value relative to its mean:

Classification	Local value	Neighbors' values	Interpretation
High-High (Hot spot)	Above mean	Above mean	Cluster of high incidence
Low-Low (Cold spot)	Below mean	Below mean	Cluster of low incidence
High-Low (Outlier)	Above mean	Below mean	High-incidence municipality surrounded by low
Low-High (Outlier)	Below mean	Above mean	Low-incidence municipality surrounded by high
Not significant	—	—	$p \geq 0.05$, no significant local association

Classification was conditional on the pseudo p-value being < 0.05. Pseudo p-values were computed using the conditional permutation approach with 999 random permutations, as implemented in localmoran() from the spdep package.

Standardization for Moran Scatter Plot

For the Moran scatter plot (Figure 3, Panel C), both the incidence rate and its spatial lag were mean-centered:

$$z_i = x_i - \bar{x} \quad \text{(standardized local value)}$$

$$wz_i = \mathbf{W}x_i - \mathbf{W}\bar{x} \quad \text{(standardized spatial lag)}$$

The four quadrants of the scatter plot correspond to the four LISA cluster types, with the regression line slope equal to the global Moran's *I*.

Multi-Level Analysis Rationale

Scale Effect

National-level LISA analysis is sensitive to scale effects. When a single state (Chihuahua, ~65% of cases) dominates the case distribution, municipalities in other states with moderate case counts appear as non-significant due to the national-scale comparison. This produces the expected finding that nearly all hot spots concentrate in the dominant cluster.

To address this limitation, we implemented two complementary approaches:

- **Wave-stratified analysis** re-estimates LISA using wave-specific incidence rates (Wave 1 vs Wave 2), revealing how spatial clustering patterns shift between epidemic phases. A decline in Moran's *I* between waves indicates geographic dispersal of transmission.
- **State-level analysis** estimates Moran's *I* and LISA within each state's municipalities only, using a state-specific spatial weights matrix. This detects intra-state clustering invisible at the national scale. States were included if they had ≥100 confirmed cases and ≥20 municipalities to ensure sufficient statistical power for the spatial weights calculation.

Minimum Requirements for State-Level Analysis

Criterion	Threshold	Rationale
Confirmed cases	≥ 100	Sufficient variability in incidence rates
Municipalities	≥ 20	Minimum for stable spatial weights matrix
Municipalities with cases	≥ 5	Prevent trivial results

Spatial Diffusion Analysis

The spatial diffusion pattern was assessed using linear regression of the epidemiological week of first case arrival against distance from the outbreak epicenter (Cuauhtémoc, Chihuahua):

$$\text{EW\_first}_i = \beta_0 + \beta_1 \times \text{Distance}_i + \varepsilon_i$$

where:

- EW_first_i is the epidemiological week of the first confirmed case in municipality *i*
- Distance_i is the Euclidean distance (km) from the centroid of municipality *i* to the centroid of Cuauhtémoc, Chihuahua
- β_1 represents the rate of spatial spread (weeks per km)

The propagation velocity was estimated as:

$$\text{Velocity (km/week)} = 1 \ / \ \beta_1 \times 7$$

A significant positive relationship ($p < 0.05$) indicates a contagious diffusion process consistent with radial spread from the epicenter, as opposed to hierarchical diffusion (urban-to-urban) or random spatial patterns. Distance was computed between municipality centroids using st_distance() from the sf package, with coordinates in the default CRS (WGS84, EPSG:4326). Point sizes in the scatter plot (Figure 3, Panel E) are proportional to the number of cases in each municipality.

Supplementary Figure S2. Vaccination coverage and vaccine effectiveness during the measles outbreak, Mexico 2025–2026.

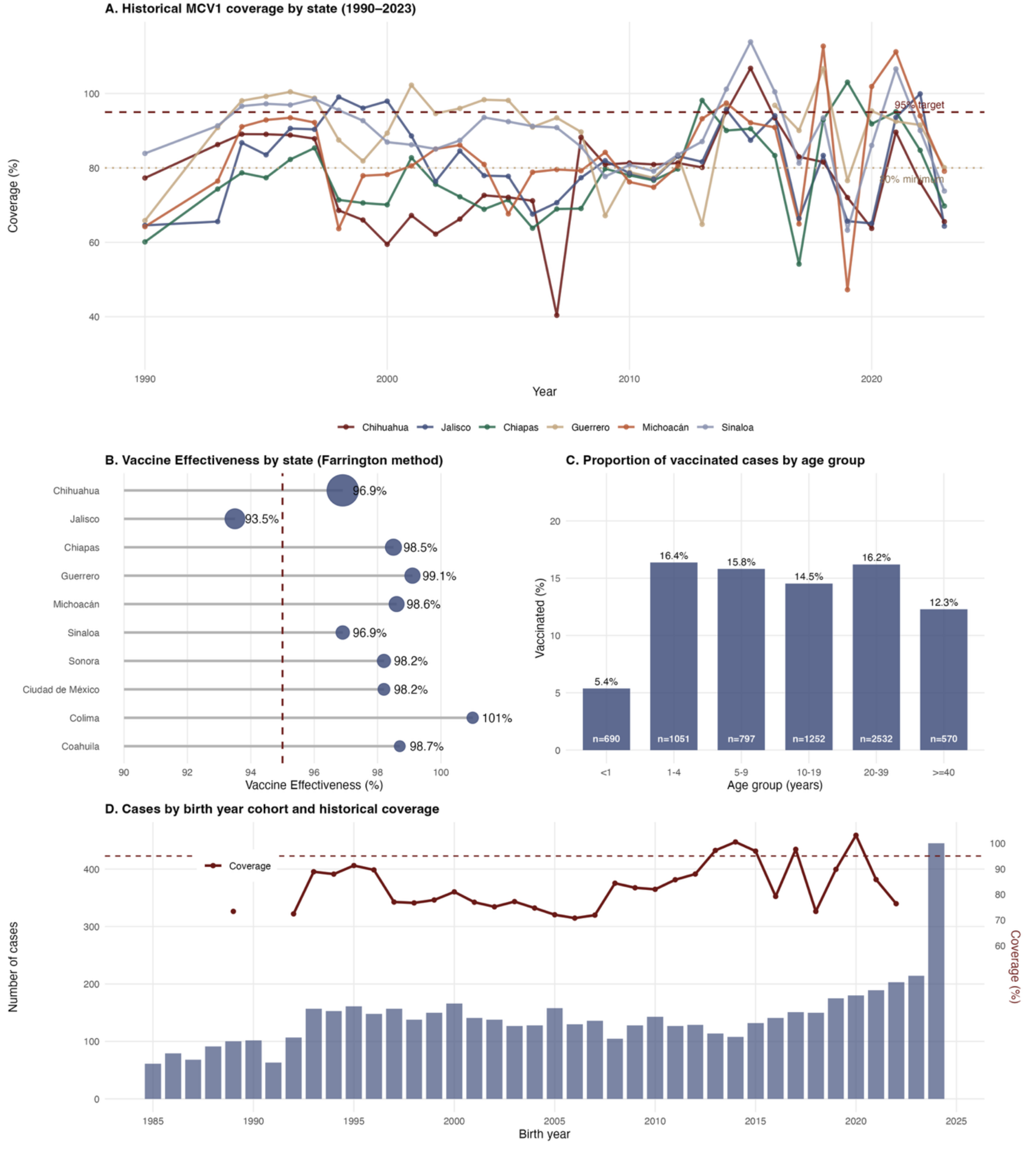

Data sources: CENSIA/CNI (coverage 1990–2023), EFEs/DGE (cases 2025–2026, N = 6,892). VE calculated using Farrington screening method.
States shown: Top 6 by case count. Dashed line = 95% coverage target.

(A) Historical MCV1 coverage by state (1990–2023) for the six most-affected states; red dashed line = 95% herd immunity threshold, orange dotted line = 80% minimum. Chihuahua and Jalisco consistently fell below the 95% target. **(B)** Vaccine effectiveness (VE) by state using the Farrington screening method (Supplementary Note S3); states with ≥50 confirmed cases shown. Point size reflects case count. **(C)** Proportion of vaccinated cases by age group, with lowest proportion (<1 year, 5.4%) reflecting pre-vaccination age. **(D)** Cases by birth-year cohort overlaid with historical national MCV1 coverage (red line), showing accumulation of susceptibles across birth cohorts with coverage gaps.

Background and Rationale

Vaccine effectiveness (VE) measures the proportional reduction in disease risk among vaccinated compared to unvaccinated individuals under real-world conditions. Unlike vaccine efficacy (measured in controlled clinical trials), VE accounts for programmatic factors including cold-chain integrity, vaccination timing, and population-level immunity.

We estimated VE using the **Farrington screening method** (Farrington, 1993), a widely used ecological approach designed for outbreak investigations where individual-level population data are unavailable. The method requires only two inputs: the proportion of cases that are vaccinated (PCV) and the proportion of the population that is vaccinated (PPV). This approach has been applied in recent measles outbreak investigations in the United States (Patel et al., 2020, MMWR; Rosen et al., 2023, Pediatrics) and globally (Orenstein et al., 1985, 1988).

Farrington Screening Method

The screening method estimates VE from the relationship between the vaccination status of cases and the background vaccination coverage of the source population:

$$VE = 1 - OR_{screening}$$

$$\text{where: } OR_{screening} = [PCV / (1 - PCV)] \times [(1 - PPV) / PPV]$$

This simplifies to the standard Farrington formula:

$$VE = 1 - [(PCV \times (1 - PPV)) / (PPV \times (1 - PCV))]$$

Parameters:

Symbol	Definition	Source
PCV	Proportion of cases vaccinated	SINAVE/DGE surveillance data (VACUNACION = 1)
PPV	Population proportion vaccinated	Mean MCV1 coverage 2014-2023 (CENSIA/CNI)
VE	Vaccine effectiveness	Derived

Derivation

The screening method is derived from the case-coverage (or case-population) design. If we define:

- a = vaccinated cases
- b = unvaccinated cases
- PCV = a / (a + b)
- PPV = population vaccination coverage

Then the odds ratio comparing vaccination odds among cases vs. population is:

$$OR = [a / b] / [PPV / (1 - PPV)]$$
$$= [PCV / (1 - PCV)] / [PPV / (1 - PPV)]$$

Under the assumptions that the vaccine does not affect disease ascertainment and that coverage data are representative, this OR approximates the relative risk, and:

$$VE = 1 - OR$$

Assumptions

The Farrington screening method assumes:

1. **Representative case ascertainment:** Vaccination status is ascertained equally in vaccinated and unvaccinated cases (no differential reporting bias).
2. **Accurate coverage data:** PPV reflects the true vaccination coverage of the source population for the relevant age cohorts.
3. **Homogeneous mixing:** Vaccinated and unvaccinated individuals have equal exposure risk (no confounding by exposure probability).
4. **Stable coverage:** PPV is approximately constant over the relevant time period.
5. **Complete ascertainment of vaccination status:** Misclassification of vaccination status is minimal.

In outbreak settings, assumption 3 may be violated if unvaccinated individuals cluster geographically or socially (as in Mennonite or indigenous communities), which can lead to VE underestimation. We address this by computing state-specific estimates where clustering is more homogeneous.

S3.3 Confidence Intervals: Orenstein Method

Confidence intervals for VE were calculated using the **Orenstein method** (Orenstein et al., 1985, 1988), which applies the normal approximation to the log-odds ratio.

Formulas

Step 1: Calculate the screening odds ratio

$$OR = [PCV / (1 - PCV)] / [PPV / (1 - PPV)]$$

Step 2: Standard error of the natural logarithm of OR

$$SE(\ln OR) = \sqrt{(1/a + 1/b)}$$

where a = number of vaccinated cases and b = number of unvaccinated cases.

This standard error is derived from the binomial variance of PCV, treating PPV as a known population parameter (not subject to sampling variability).

Step 3: 95% confidence interval for the odds ratio

$$OR_{lower} = \exp[\ln(OR) - 1.96 \times SE(\ln OR)]$$
$$OR_{upper} = \exp[\ln(OR) + 1.96 \times SE(\ln OR)] \qquad \text{(Equation 5)}$$

Step 4: Convert to VE scale

Since VE = 1 − OR, the confidence limits are inverted:

$$VE_{lower} = 1 - OR_{upper}$$
$$VE_{upper} = 1 - OR_{lower}$$

Interpretation

The Orenstein CI is conservative because it treats PPV as fixed. In practice, PPV is estimated from administrative coverage data, which introduces additional uncertainty not captured in the CI. However, the large population denominators (state or national level) make this additional variance negligible.

Additional Epidemiological Metrics

Relative Risk (RR)

The relative risk of measles among unvaccinated vs. vaccinated individuals was estimated from the case-population framework:

$$RR = P(\text{case} \mid \text{unvac}) / P(\text{case} \mid \text{vac})$$
$$\text{Estimated as: } RR = [p_{unvac\_cases} / p_{unvac\_pop}] / [(1 - p_{unvac\_cases}) / (1 - p_{unvac\_pop})]$$

Symbol	Definition	Value
p_{unvac_cases}	Proportion of cases that are unvaccinated	85.5% (5,895/6,892)
p_{unvac_pop}	Proportion of population unvaccinated	10% (1 − PPV)

Population Attributable Fraction (PAF)

The PAF represents the proportion of cases that would theoretically be prevented if the entire population were vaccinated:

$$PAF = p_{unvac\_cases} \times (RR - 1) / [p_{unvac\_cases} \times (RR - 1) + 1]$$

A PAF approaching 100% indicates that nearly all cases are attributable to non-vaccination.

Supplementary Figure S3. Measles Virus Genotype Distribution in Mexico, Genomic surveillance data from GenBank (2011–2023) and SSA Epidemiological Bulletins (2025)

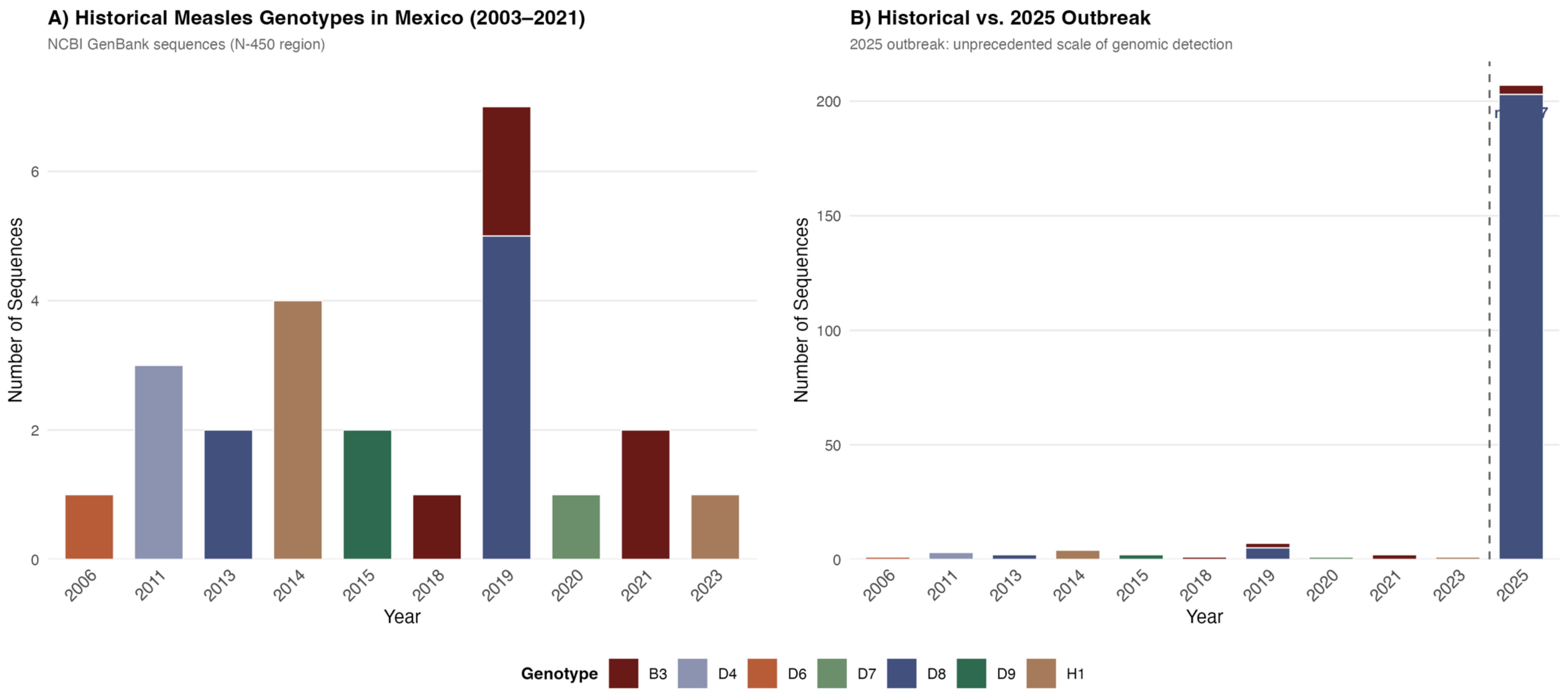

Supplementary Table S1. Data dictionary of variables from linked national open-access databases used in the analysis.

Variable	Type	Data Source	Description
EDAD_ANOS	Numeric (continuous)	EFEs - Epidemiological Surveillance	Patient age in completed years
EDAD_MESES	Numeric (continuous)	EFEs - Epidemiological Surveillance	Patient age in months (for <1 year old)
EDAD_DIAS	Numeric (continuous)	EFEs - Epidemiological Surveillance	Patient age in days (for <31 days old)
SEXO	Categorical (binary)	EFEs - Epidemiological Surveillance	Patient biological sex (1=Male, 2=Female)
HABLA_LENGUA_INDIG	Categorical (binary)	EFEs - Epidemiological Surveillance	Patient speaks an indigenous language (1=Yes, 2=No)
INDIGENA	Categorical (binary)	EFEs - Epidemiological Surveillance	Patient self-identifies as indigenous (1=Yes, 2=No)
ENTIDAD_RES	Categorical (nominal)	EFEs - Epidemiological Surveillance	State of residence (2-digit code)
MUNICIPIO_RES	Categorical (nominal)	EFEs - Epidemiological Surveillance	Municipality of residence (3-digit code)
ENTIDAD_UM_NOTIF	Categorical (nominal)	EFEs - Epidemiological Surveillance	State of notification (healthcare facility location)
MUNICIPIO_UM_NOTIF	Categorical (nominal)	EFEs - Epidemiological Surveillance	Municipality of notification (healthcare facility location)
INSTITUCION_NOTIF	Categorical (nominal)	EFEs - Epidemiological Surveillance	Healthcare institution reporting the case (IMSS, ISSSTE, SSA, etc.)
VACUNACION	Categorical (binary)	EFEs - Epidemiological Surveillance	Documented measles/rubella vaccination status (SRP or SR) in health card (1=Yes, 2=No)
EXANTEMA	Categorical (binary)	EFEs - Epidemiological Surveillance	Patient presented exanthema/rash (1=Yes, 2=No)
FIEBRE	Categorical (binary)	EFEs - Epidemiological Surveillance	Patient presented fever (1=Yes, 2=No)
COMPLICACIONES	Categorical (binary)	EFEs - Epidemiological Surveillance	Patient developed complications (1=Yes, 2=No)
DEFUNCION	Categorical (binary)	EFEs - Epidemiological Surveillance	Patient died (1=Yes, 2=No)
DIAGNOSTICO	Categorical (nominal)	EFEs - Epidemiological Surveillance	Final diagnosis (1=Measles confirmed, 2=Rubella, 3=Discarded)
CRITERIO_DIAGNOSTICO	Categorical (nominal)	EFEs - Epidemiological Surveillance	Diagnostic criteria used for case confirmation (Laboratory, Clinical-epidemiological)
FECHA_DIAGNOSTICO	Date	EFEs - Epidemiological Surveillance	Date of diagnosis confirmation by InDRE reference laboratory
FECHA_INICIO_SIGNOS	Date	EFEs - Epidemiological Surveillance	Date of symptom onset
SEMANA_EPI	Numeric (integer)	EFEs - Epidemiological Surveillance	Epidemiological week of symptom onset
ORIGEN_CASO	Categorical (nominal)	EFEs - Epidemiological Surveillance	Transmission origin (Indigenous, Imported, Import-related)
POB_TOT	Numeric (continuous)	CONAPO - Marginalization Index 2020	Total population of the municipality
ANALF	Numeric (percentage)	CONAPO - Marginalization Index 2020	Percentage of population aged 15+ who are illiterate
SBASC	Numeric (percentage)	CONAPO - Marginalization Index 2020	Percentage of population aged 15+ without complete basic education
OVSDE	Numeric (percentage)	CONAPO - Marginalization Index 2020	Percentage of occupied private dwellings without drainage or toilet
OVSEE	Numeric (percentage)	CONAPO - Marginalization Index 2020	Percentage of occupied private dwellings without electricity
OVSAE	Numeric (percentage)	CONAPO - Marginalization Index 2020	Percentage of occupied private dwellings without piped water
OVPT	Numeric (percentage)	CONAPO - Marginalization Index 2020	Percentage of occupied private dwellings with dirt floor
VHAC	Numeric (percentage)	CONAPO - Marginalization Index 2020	Percentage of private dwellings with overcrowding (>2.5 persons per room)
PL.5000	Numeric (percentage)	CONAPO - Marginalization Index 2020	Percentage of population living in localities with <5,000 inhabitants (rurality indicator)
PO2SM	Numeric (percentage)	CONAPO - Marginalization Index 2020	Percentage of employed population earning ≤2 minimum wages
IM_2020	Numeric (continuous)	CONAPO - Marginalization Index 2020	Marginalization Index 2020 - composite score based on principal component analysis
GM_2020	Categorical (ordinal)	CONAPO - Marginalization Index 2020	Marginalization Degree 2020 (Muy bajo, Bajo, Medio, Alto, Muy alto)
IMN_2020	Numeric (continuous)	CONAPO - Marginalization Index 2020	Normalized Marginalization Index 2020 (0-1 scale)
VIV_REM	Numeric (percentage)	CONAPO - Migration Intensity Index 2020	Percentage of households receiving remittances from abroad
VIV_EMIG	Numeric (percentage)	CONAPO - Migration Intensity Index 2020	Percentage of households with emigrants to USA in previous 5 years
VIV_CIRC	Numeric (percentage)	CONAPO - Migration Intensity Index 2020	Percentage of households with circular migrants to USA
VIV_RET	Numeric (percentage)	CONAPO - Migration Intensity Index 2020	Percentage of households with return migrants from USA
IIM_DP2	Numeric (continuous)	CONAPO - Migration Intensity Index 2020	Migration Intensity Index (DP2 method) - composite score
GIM_DP2	Categorical (ordinal)	CONAPO - Migration Intensity Index 2020	Migration Intensity Degree (Nulo, Muy bajo, Bajo, Medio, Alto, Muy alto)

Variable	Type	Data Source	Description
poblacion	Numeric (continuous)	CONEVAL - Social Lag Index 2020	Total municipality population
i_analf	Numeric (percentage)	CONEVAL - Social Lag Index 2020	Percentage of population aged 15+ who are illiterate
i_asistesc	Numeric (percentage)	CONEVAL - Social Lag Index 2020	Percentage of population aged 6-14 not attending school
i_edbasinc	Numeric (percentage)	CONEVAL - Social Lag Index 2020	Percentage of population aged 15+ with incomplete basic education
i_sdsalud	Numeric (percentage)	CONEVAL - Social Lag Index 2020	Percentage of population without health services entitlement
i_ptierra	Numeric (percentage)	CONEVAL - Social Lag Index 2020	Percentage of dwellings with dirt floor
i_nosan	Numeric (percentage)	CONEVAL - Social Lag Index 2020	Percentage of dwellings without toilet or latrine
i_noagua	Numeric (percentage)	CONEVAL - Social Lag Index 2020	Percentage of dwellings without piped water from public network
i_nodren	Numeric (percentage)	CONEVAL - Social Lag Index 2020	Percentage of dwellings without drainage
i_noelec	Numeric (percentage)	CONEVAL - Social Lag Index 2020	Percentage of dwellings without electricity
i_nolav	Numeric (percentage)	CONEVAL - Social Lag Index 2020	Percentage of dwellings without washing machine
i_noref	Numeric (percentage)	CONEVAL - Social Lag Index 2020	Percentage of dwellings without refrigerator
irs	Numeric (continuous)	CONEVAL - Social Lag Index 2020	Social Lag Index (composite score from PCA) - higher values indicate greater lag
grs	Categorical (ordinal)	CONEVAL - Social Lag Index 2020	Social Lag Degree (Muy bajo, Bajo, Medio, Alto, Muy alto)
lugar	Numeric (ordinal)	CONEVAL - Social Lag Index 2020	National ranking position (1=highest lag among 2,469 municipalities)
UP_TOTAL	Numeric (count)	INEGI - Agricultural Census 2022	Total active agricultural production units in the municipality
UP_NOREMUN	Numeric (count)	INEGI - Agricultural Census 2022	Production units with unpaid workers
UP_REMUN	Numeric (count)	INEGI - Agricultural Census 2022	Production units with paid workers
UP_JORNAL	Numeric (count)	INEGI - Agricultural Census 2022	Production units employing day laborers (jornaleros)
UP_JORNAL_HOM	Numeric (count)	INEGI - Agricultural Census 2022	Production units employing male day laborers
MO_JORNAL_MUJ	Numeric (count)	INEGI - Agricultural Census 2022	Female day laborers (jornaleras) employed
UP_MAS6	Numeric (count)	INEGI - Agricultural Census 2022	Production units with workers employed >6 months/year
UP_MENOS6	Numeric (count)	INEGI - Agricultural Census 2022	Production units with workers employed <6 months/year (seasonal)
CLUES	Categorical (identifier)	DGIS - Hospital Discharges (SAEH) 2025	Unique health facility identifier code
FECHA_INGRESO	Date	DGIS - Hospital Discharges (SAEH) 2025	Hospital admission date
FECHA_EGRESO	Date	DGIS - Hospital Discharges (SAEH) 2025	Hospital discharge date
DIAS_ESTA	Numeric (continuous)	DGIS - Hospital Discharges (SAEH) 2025	Length of hospital stay in days
EDAD	Numeric (continuous)	DGIS - Hospital Discharges (SAEH) 2025	Patient age (numeric value)
CVEEDAD	Categorical (ordinal)	DGIS - Hospital Discharges (SAEH) 2025	Age unit code (4=years, 5=months, etc.)
GRUPO_EDAD	Categorical (ordinal)	DGIS - Hospital Discharges (SAEH) 2025	Age group category (e.g., <1 year, 1-4 years, 5-9 years)
SEXO_SAEH	Categorical (binary)	DGIS - Hospital Discharges (SAEH) 2025	Patient biological sex (1=Male, 2=Female)
INDIGENA_SAEH	Categorical (binary)	DGIS - Hospital Discharges (SAEH) 2025	Patient identifies as indigenous (1=Yes, 2=No)
HABLA_LENGUA	Categorical (binary)	DGIS - Hospital Discharges (SAEH) 2025	Patient speaks indigenous language (1=Yes, 2=No)
AFECPRIN	Categorical (ICD-10)	DGIS - Hospital Discharges (SAEH) 2025	Primary diagnosis ICD-10 code (B05.x for measles)
COMPLICACION_SAEH	Categorical (nominal)	DGIS - Hospital Discharges (SAEH) 2025	Type of complication (Pneumonia, Encephalitis, etc.)
CON_COMPLICACION	Categorical (binary)	DGIS - Hospital Discharges (SAEH) 2025	Patient had complications (0=No, 1=Yes)
DIAG_INI	Categorical (ICD-10)	DGIS - Hospital Discharges (SAEH) 2025	Initial diagnosis ICD-10 code at admission
MOTEGRE	Categorical (nominal)	DGIS - Hospital Discharges (SAEH) 2025	Discharge reason (1=Recovery, 2=Improvement, 3=Transfer, 4=Voluntary, 5=Death)
DEFUNCION_SAEH	Categorical (binary)	DGIS - Hospital Discharges (SAEH) 2025	In-hospital death (0=No, 1=Yes)
YEAR	Numeric (year)	CENSIA - BIO-SIS 1990-2023	Calendar year of coverage measurement
ENTIDAD	Categorical (nominal)	CENSIA - BIO-SIS 1990-2023	State/federal entity name
COB_SRP1	Numeric (percentage)	CENSIA - BIO-SIS 1990-2023	Coverage of first dose MMR vaccine (SRP1) in children 1 year old
COB_SRP2	Numeric (percentage)	CENSIA - BIO-SIS 1990-2023	Coverage of second dose MMR vaccine (SRP2) in children 6 years old
POB_MIT_ANO	Numeric (continuous)	CONAPO - Population Projections 2020-2070	Mid-year population estimate by municipality
POB_EDAD	Numeric (continuous)	CONAPO - Population Projections 2020-2070	Population by age group for incidence rate denominators
CVE_MUN	Categorical (identifier)	Derived	5-digit municipal code (2-digit state + 3-digit municipality)
PCT_JORNAL	Numeric (percentage)	Derived from Agricultural Census	Percentage of agricultural units employing day laborers: (UP_JORNAL / UP_TOTAL) × 100
PCT_ESTACIONAL	Numeric (percentage)	Derived from Agricultural Census	Percentage of agricultural units with seasonal workers: (UP_MENOS6 / UP_TOTAL) × 100

Variable	Type	Data Source	Description
TERCIL_JORNAL	Categorical (ordinal)	Derived	Day laborer percentage tertiles: T1-Low (<33rd percentile), T2-Medium, T3-High (>66th percentile)
ALTA_MIGRACION	Categorical (binary)	Derived from Migration Index	High migration intensity municipality: GIM_DP2 = 'Alto' or 'Muy alto'
ALTA_MARGINACION	Categorical (binary)	Derived from Marginalization Index	High marginalization municipality: GM_2020 = 'Alto' or 'Muy alto'
ALTO_REZAGO	Categorical (binary)	Derived from Social Lag Index	High social lag municipality: grs = 'Alto' or 'Muy alto'
TIENE_CASO	Categorical (binary)	Derived from EFEs	Municipality has at least one confirmed measles case (TRUE/FALSE)
TASA	Numeric (continuous)	Derived	Municipal incidence rate per 100,000 population

Supplementary Table S2. Demographic and clinical characteristics of confirmed measles cases by age group, measles outbreak, Mexico, 2025-2026.

**Characteristic**	**<1 year** N = 690[1]	**1-4 years** N = 1051[1]	**5-9 years** N = 797[1]	**10-19 years** N = 1252[1]	**20-39 years** N = 2532[1]	**≥40 years** N = 570[1]	**Overall** N = 6892[1]	**p-value**[2]
**Sex**								<0.001
Female	342 (49.6%)	518 (49.3%)	428 (53.7%)	662 (52.9%)	1166 (46.1%)	266 (46.7%)	3382 (49.1%)	
Male	348 (50.4%)	533 (50.7%)	369 (46.3%)	590 (47.1%)	1366 (53.9%)	304 (53.3%)	3510 (50.9%)	
**Vaccination status**								<0.001
Unvaccinated	653 (94.6%)	879 (83.6%)	671 (84.2%)	1070 (85.5%)	2122 (83.8%)	500 (87.7%)	5895 (85.5%)	
Vaccinated	37 (5.4%)	172 (16.4%)	126 (15.8%)	182 (14.5%)	410 (16.2%)	70 (12.3%)	997 (14.5%)	
**Indigenous status**	229 (33.2%)	414 (39.4%)	288 (36.1%)	490 (39.1%)	509 (20.1%)	73 (12.8%)	2003 (29.1%)	<0.001
**Case origin**								<0.001
Import-related	377 (54.6%)	529 (50.3%)	356 (44.7%)	640 (51.1%)	1692 (66.8%)	399 (70.0%)	3993 (57.9%)	
Imported	29 (4.2%)	60 (5.7%)	42 (5.3%)	70 (5.6%)	43 (1.7%)	11 (1.9%)	255 (3.7%)	
Unknown source	284 (41.2%)	462 (44.0%)	399 (50.1%)	542 (43.3%)	797 (31.5%)	160 (28.1%)	2644 (38.4%)	
**Complications**	244 (35.4%)	306 (29.1%)	137 (17.2%)	144 (11.5%)	189 (7.5%)	49 (8.6%)	1069 (15.5%)	<0.001
**Death**	4 (0.6%)	9 (0.9%)	3 (0.4%)	3 (0.2%)	3 (0.1%)	3 (0.5%)	25 (0.4%)	0.012

[1]n (%)

[2]Pearson's Chi-squared test; Fisher's exact test

Supplementary Table S3. Demographic and clinical characteristics of confirmed measles cases by complication status, measles outbreak, Mexico, 2025-2026.

Characteristic	No Complications N = 5823[1]	With Complications N = 1069[1]	Overall N = 6,892[1]	p-value[2]
**Age, years**	19 (6, 30)	4 (1, 18)	17 (4, 29)	<0.001
**Age group**				<0.001
<1 year	446 (7.7%)	244 (22.8%)	690 (10.0%)	
1-4 years	745 (12.8%)	306 (28.6%)	1,051 (15.2%)	
5-9 years	660 (11.3%)	137 (12.8%)	797 (11.6%)	
10-19 years	1,108 (19.0%)	144 (13.5%)	1,252 (18.2%)	
20-39 years	2,343 (40.2%)	189 (17.7%)	2,532 (36.7%)	
≥40 years	521 (8.9%)	49 (4.6%)	570 (8.3%)	
**Sex**				>0.9
Female	2,858 (49.1%)	524 (49.0%)	3,382 (49.1%)	
Male	2,965 (50.9%)	545 (51.0%)	3,510 (50.9%)	
**Vaccination status**				<0.001
Unvaccinated	4,907 (84.3%)	988 (92.4%)	5,895 (85.5%)	
Vaccinated	916 (15.7%)	81 (7.6%)	997 (14.5%)	
**Indigenous status**	1,455 (25.0%)	548 (51.3%)	2,003 (29.1%)	<0.001
**Case origin**				0.004
Import-related	3,415 (58.6%)	578 (54.1%)	3,993 (57.9%)	
Imported	222 (3.8%)	33 (3.1%)	255 (3.7%)	
Unknown source	2,186 (37.5%)	458 (42.8%)	2,644 (38.4%)	

[1]Median (Q1, Q3); n (%)

[2]Wilcoxon rank sum test; Pearson's Chi-squared test; Fisher's exact test

Supplementary Table S4. Demographic and clinical characteristics of confirmed measles cases by outbreak phase, measles outbreak, Mexico, 2025-2026.

**Characteristic**	**Introduction (Wk 8-16)** N = 647[1]	**Peak (Wk 17-28)** N = 2,770[1]	**Decline (Wk 29-40)** N = 1,398[1]	**Late (Wk 41-53)** N = 1,336[1]	**Resurgence (Wk 54+)** N = 741[1]	**Overall** N = 6,892[1]
**Age, years**	19 (6, 30)	23 (6, 32)	13 (2, 25)	11 (4, 22)	15 (6, 28)	17 (4, 29)
**Age group**						
<1 year	44 (6.8%)	263 (9.5%)	198 (14.2%)	136 (10.2%)	49 (6.6%)	690 (10.0%)
1-4 years	89 (13.8%)	343 (12.4%)	259 (18.5%)	251 (18.8%)	109 (14.7%)	1,051 (15.2%)
5-9 years	79 (12.2%)	213 (7.7%)	155 (11.1%)	233 (17.4%)	117 (15.8%)	797 (11.6%)
10-19 years	113 (17.5%)	388 (14.0%)	271 (19.4%)	322 (24.1%)	158 (21.3%)	1,252 (18.2%)
20-39 years	267 (41.3%)	1,262 (45.6%)	423 (30.3%)	327 (24.5%)	253 (34.1%)	2,532 (36.7%)
≥40 years	55 (8.5%)	301 (10.9%)	92 (6.6%)	67 (5.0%)	55 (7.4%)	570 (8.3%)
**Sex**						
Female	335 (51.8%)	1,293 (46.7%)	718 (51.4%)	659 (49.3%)	377 (50.9%)	3,382 (49.1%)
Male	312 (48.2%)	1,477 (53.3%)	680 (48.6%)	677 (50.7%)	364 (49.1%)	3,510 (50.9%)
**Vaccination status**						
Unvaccinated	580 (89.6%)	2,383 (86.0%)	1,209 (86.5%)	1,111 (83.2%)	612 (82.6%)	5,895 (85.5%)
Vaccinated	67 (10.4%)	387 (14.0%)	189 (13.5%)	225 (16.8%)	129 (17.4%)	997 (14.5%)
**Indigenous status**	5 (0.8%)	504 (18.2%)	763 (54.6%)	613 (45.9%)	118 (15.9%)	2,003 (29.1%)
**Complications**	96 (14.8%)	399 (14.4%)	351 (25.1%)	160 (12.0%)	63 (8.5%)	1,069 (15.5%)
**Death**	2 (0.3%)	10 (0.4%)	12 (0.9%)	1 (0.1%)	0 (0.0%)	25 (0.4%)

[1]Median (Q1, Q3); n (%). [2]Kruskal-Wallis rank sum test; Pearson's Chi-squared test; Fisher's exact test

Supplementary Table S5. Summary of effective reproduction number (Rt) estimates by geographic region and extended methodology, measles outbreak, Mexico, 2025-2026.

	Peak Transmission			Duration			Most Recent Estimate		
**Region**	**Peak Rt***	**95% CrI**	**Date of Peak**	**Date Rt First <1**	**Days Rt >1**	**Days Rt <1**	**Last Reliable Rt**	**Last Date**	**Status**
Michoacan	74.9	48.4-106.9	2025-09-28	2025-10-08	62	43	38.96	2026-01-16	Active
Jalisco	64.0	45.9-85	2025-10-02	2025-10-12	76	37	3.57	2026-01-19	Active
Guerrero	60.0	31-98.4	2025-08-08	2025-09-13	75	30	13.14	2026-01-19	Active
Sinaloa	40.0	17.3-72.1	2025-07-13	2025-12-29	33	12	4.33	2026-01-20	Active
Chiapas	17.4	14.8-20.1	2026-01-17	Not reached	31	8	15.27	2026-01-18	Active
Chihuahua	13.0	8.9-17.8	2025-03-17	2025-05-06	142	151	0.38	2025-12-28	Controlled
National	12.1	8.5-16.4	2025-03-15	2025-05-08	183	136	2.81	2026-01-20	Active

Rt estimated using EpiEstim (Cori et al., 2013) with parametric serial interval (mean: 11.7 days, SD: 2.0 days; Lessler et al., 2009). 7-day sliding window with Bayesian inference.
Rt estimates suppressed when 7-day rolling case count < 5 to avoid artifacts from sparse incidence data.
**Peak Rt values for states with late epidemic onset (Michoacán, Jalisco, Guerrero, Sinaloa) reflect introduction-phase artifacts when small case clusters follow periods of zero incidence; these values should be interpreted with caution.*
'Controlled' = last reliable Rt < 1; 'Active' = last reliable Rt >= 1.
Source: SINAVE/DGE, Secretaria de Salud. Data through EW 3/2026 (N = 6,892 cases).

The instantaneous reproduction number (Rt) was estimated using the framework developed by Cori et al. (2013), implemented via the EpiEstim package (v2.2) in R. This method defines Rt as the ratio of current incidence to the aggregate infectiousness of previously infected individuals.

Instantaneous Reproduction Number

The Rt at time t is defined as:

$$Rt = It \;/\; \Lambda t$$

Where:

- It: Incidence (new cases) at time t.
- Λt: Total infectiousness at time t, computed as:

$$\Lambda t = \Sigma s{=}1t\ It{-}s\ ws$$

where ws is the probability mass function (PMF) of the serial interval distribution at lag s.

Bayesian Estimation

EpiEstim utilizes a Bayesian framework with conjugate Gamma priors:

Prior: $R_t \sim \text{Gamma}(a, b)$

Posterior: $R_t \mid I_t \sim \text{Gamma}(a + \Sigma I_s,\ 1/(1/b + \Sigma\Lambda_s))$

An uninformative prior was used ($a = 1$, $b = 5$). The posterior mean of R_t over an estimation window $[t_{start}, t_{end}]$ is:

$$E[R_t] = (a + \Sigma I_s) \;/\; (1/b + \Sigma\Lambda_s)$$

To ensure stability against daily reporting fluctuations, a 7-day sliding window (τ = 7) was applied:

$$R_t^{\tau} = (a + \Sigma_{s=t-\tau+1}^{t} I_s) \;/\; (1/b + \Sigma_{s=t-\tau+1}^{t} \Lambda_s)$$

The serial interval was modeled as a Gamma distribution following Lessler et al. (2009):

**Parameter**	**Value**	**Source**
Mean (μ)	11.7 days	Lessler et al. (2009)
Std. Deviation (σ)	2.0 days	Lessler et al. (2009)
Shape ($k = \mu^2/\sigma^2$)	34.2	Derived
Rate ($\theta = \mu/\sigma^2$)	2.925	Derived

To prevent unreliable or artificially inflated Rt estimates during low-incidence periods, a suppression filter was implemented:

Criteria: If $\Sigma s{=}t{-}6t\ Is < 5$, then Rt = NA

This threshold ensures that the posterior distribution is driven by observed data rather than the prior distribution, particularly in regions with sporadic transmission (e.g., Chiapas and Sinaloa, which showed >75% suppression).

Supplementary Table S6. Spatial analysis of measles outbreak clustering and diffusion, Mexico 2025–2026. Global spatial autocorrelation (Moran's I) by analysis level (national, wave-stratified, and state-level), LISA cluster summary, wave comparison, and spatial diffusion parameters. Queen contiguity weights; 999 permutations. Hot spots defined as High-High clusters ($p < 0.05$).

Global Spatial Autocorrelation by Analysis Level

Level	Moran's I	z-score	p-value	N municipalities	Hot spots
National (full period)	0.410	35.3	<0.001	2,457	47
Wave 1 (EW 8–53/2025)	0.412	35.4	<0.001	2,457	46
Wave 2 (EW 1–3/2026)	0.170	14.8	<0.001	2,457	33
Chihuahua (state)	0.191	2.8	0.003	67	5
Jalisco (state)	0.045	1.0	0.153	125	3
Chiapas (state)	0.246	4.5	<0.001	118	11
Guerrero (state)	−0.013	0.0	0.504	81	2
Sonora (state)	0.143	2.6	0.005	72	2

LISA Cluster Summary (National)

Cluster type	Municipalities	Cases	Mean rate (per 100,000)	% of total cases
High-High (Hot spot)	47	4,441	251.6	68.4%
Low-High (Outlier)	18	1	0.2	0.0%
Not significant	2,392	2,049	1.5	31.6%

Wave Comparison*

Wave	Period	Moran's I	Hot spots	Interpretation
Wave 1	EW 8–53/2025	0.412	46	Concentrated clustering (Chihuahua epicenter)
Wave 2	EW 1–3/2026	0.170	33	Dispersed pattern (multi-state resurgence)

Spatial Diffusion**

Parameter	Value
Epicenter	Cuauhtémoc, Chihuahua
R^2	0.418
p-value	<0.001
Estimated velocity	~459 km/week
N municipalities	236 (with ≥1 case)

* The 59% reduction in Moran's *I* from Wave 1 to Wave 2 (0.412 → 0.170), accompanied by a geographic shift in hot spot locations, is consistent with a transition from a single-epicenter outbreak to a multi-focal resurgence pattern.

** The moderate R^2 (0.418) indicates that distance from the epicenter explains approximately 42% of the variance in timing of first case arrival, consistent with a predominantly contagious diffusion process with some hierarchical (long-distance) jumps.

Supplementary Table S7. Comparison of logistic regression models for municipal measles case presence (≥1 confirmed case), Mexico 2025–2026.

	Model 1 (base)			Model 2 (+ school non-attendance)		
**Variable**	**OR**	**95% CI**	**p**	**OR**	**95% CI**	**p**
Intercept	0.32	0.25–0.40	<0.001	0.07	0.05–0.10	<0.001
**Marginalization degree (ref: Very low)**						
Low marginalization	0.58	0.39–0.85	0.006	0.42	0.28–0.62	<0.001
Medium marginalization	0.43	0.26–0.68	<0.001	0.31	0.19–0.49	<0.001
High marginalization	0.38	0.22–0.63	<0.001	0.23	0.13–0.39	<0.001
Very high marginalization	1.89	1.13–3.14	0.014	0.65	0.36–1.15	0.144
Rural (>50% in loc. <5,000)	0.41	0.29–0.59	<0.001	0.42	0.29–0.60	<0.001
Seasonal agric. workers (mun. >50%)	0.80	0.60–1.06	0.124	—		
High migration intensity	0.68	0.44–1.02	0.069	0.65	0.42–0.98	0.049
School non-attendance 6–14 yr (%)	—			1.26	1.21–1.31	<0.001
Seasonal workers (%)	—			1.02	1.00–1.04	0.074
**Model diagnostics**						
N (events / non-events)	2454 (256/2198)			2454 (256/2198)		
Parameters (EPV)	8 (32.0)			9 (28.4)		
AIC	1521.6			1380.0		
BIC	1568.0			1432.2		
McFadden pseudo-R^2	0.0828			0.1703		
Nagelkerke pseudo-R^2	0.1105			0.2208		
Hosmer-Lemeshow χ^2 (p)	7.32 (0.502)			7.10 (0.526)		
ROC AUC	0.716			0.795		
Sensitivity / Specificity	69.5%/63.0%			75.0%/69.2%		
Max VIF (GVIF^1/2Df)	1.32			1.27		

Model 1: GM_2020 + rural + seasonal agric. workers (>50%) + high migration. Model 2: GM_2020 + rural + school non-attendance 6–14 yr + high migration + seasonal workers (%). Ref: very low marginalization. EPV = events per variable. VIF as GVIF^(1/(2*Df)).

Model diagnostics include AIC, BIC, McFadden and Nagelkerke pseudo-R^2, Hosmer-Lemeshow goodness-of-fit test, ROC AUC, sensitivity/specificity, and variance inflation factors.

Supplementary Table S8. Social determinants of measles cases stratified by state, Mexico 2025–2026. Analysis restricted to states with ≥100 confirmed cases (Chihuahua, Jalisco, Guerrero, Chiapas, Sonora, Sinaloa).

**Variable**	**Overall** N = 6,365[1]	**Chihuahua** N = 4,497[1]	**Jalisco** N = 914[1]	**Guerrero** N = 256[1]	**Michoacán** N = 240[1]	**Chiapas** N = 346[1]	**Sonora** N = 112[1]	**p-value**[2]
Age (years)	17.0 (4.0, 29.0)	20.0 (4.0, 31.0)	12.0 (5.0, 23.0)	7.0 (3.0, 18.0)	10.0 (3.5, 20.0)	20.0 (7.0, 30.0)	14.0 (4.0, 24.0)	**<0.001**
Unvaccinated	5,467 (86%)	3,911 (87%)	710 (78%)	220 (86%)	216 (90%)	317 (92%)	93 (83%)	**<0.001**
Indigenous	1,851 (29%)	1,247 (28%)	164 (18%)	152 (59%)	143 (60%)	132 (38%)	13 (12%)	**<0.001**
Complications	1,041 (16%)	860 (19%)	68 (7.4%)	50 (20%)	13 (5.4%)	43 (12%)	7 (6.3%)	**<0.001**
Marginalization Index*	0.9 (0.9, 0.9)	0.9 (0.9, 0.9)	0.9 (0.9, 0.9)	0.7 (0.7, 0.8)	0.9 (0.9, 0.9)	0.9 (0.8, 0.9)	0.9 (0.9, 0.9)	**<0.001**
No health insurance (%)*	14.2 (13.1, 21.4)	13.1 (11.9, 15.4)	29.7 (28.9, 35.4)	7.9 (7.9, 28.4)	53.4 (50.5, 53.4)	41.9 (28.4, 44.7)	17.9 (15.4, 21.8)	**<0.001**
Rural population (%)*	19.3 (5.6, 38.0)	19.3 (5.6, 28.2)	3.4 (1.2, 26.0)	100.0 (44.0, 100.0)	17.4 (17.4, 33.2)	15.0 (15.0, 72.4)	8.7 (8.7, 18.4)	**<0.001**
Day laborers (%)*	32.2 (31.3, 41.8)	32.2 (31.3, 32.3)	49.1 (42.1, 53.6)	61.5 (61.5, 66.4)	75.6 (61.1, 75.6)	38.1 (31.3, 48.4)	53.3 (51.4, 62.8)	**<0.001**
High day laborer mun.*	985 (16%)	93 (2.1%)	280 (36%)	250 (98%)	217 (90%)	61 (18%)	84 (75%)	**<0.001**

[1]*Municipal-level indicator
[2]Kruskal-Wallis rank sum test; Pearson's Chi-squared test
Values are median (Q1, Q3) for continuous variables and n (%) for categorical variables.

Supplementary Table S9. Sensitivity analysis comparing the first 50 and last 50 municipalities affected during the measles outbreak, Mexico 2025–2026.

**Variable**	**First 50 mun.** (Wk 8-19) N = 4404[1]	**Last 50 mun.** (Wk 45-51) N = 89[1]	**p-value**[2]
Age (years)	20.0 (4.0, 31.0)	16.0 (8.0, 28.0)	0.645
Unvaccinated	3,821 (87%)	72 (81%)	0.146
Indigenous	1,103 (25%)	34 (38%)	**0.007**
Complications	780 (18%)	3 (3.4%)	**<0.001**
Death	22 (0.5%)	0 (0%)	>0.999
Marginalization Index (0-100)*	0.9 (0.9, 0.9)	0.9 (0.8, 0.9)	**<0.001**
Marginalization Degree*			**<0.001**
Very low	3,765 (86%)	38 (43%)	
Low	222 (5.0%)	12 (13%)	
Medium	23 (0.5%)	9 (10%)	
High	77 (1.7%)	7 (7.9%)	
Very high	316 (7.2%)	23 (26%)	
No health insurance (%)*	13.1 (12.5, 15.6)	26.6 (19.4, 32.4)	**<0.001**
Rural population <5000 (%)*	19.3 (5.6, 19.3)	44.2 (11.8, 83.2)	**<0.001**
Migration Intensity Index*	63.4 (63.4, 63.7)	64.4 (61.9, 64.9)	**<0.001**
Agricultural day laborers (%)*	32.2 (31.3, 32.3)	52.7 (44.2, 55.4)	**<0.001**
High day laborer municipality*	104 (2.4%)	44 (56%)	**<0.001**

[1]Median (Q1, Q3); n (%). *Municipal-level indicator.
[2]Wilcoxon rank sum test; Chi-squared test
Municipalities ranked by epidemiological week of first confirmed case. Values are median (Q1, Q3) for continuous variables and n (%) for categorical variables. P-values from Wilcoxon rank-sum (continuous) and Chi-squared (categorical) tests.

Supplementary Table S10. National vaccination metrics and vaccine effectiveness for the measles outbreak, Mexico 2025–2026. (a) National summary: total cases (N = 6,892), proportion vaccinated (PCV = 14.5%), population vaccination coverage (PPV = 90%), VE = 98.1% (95% CI: 98.0–98.2%), relative risk (RR = 53.2), and population attributable fraction (PAF = 97.8%). (b) VE by state for states with ≥20 confirmed cases, calculated using the Farrington screening method with 95% CI via the Orenstein method.

Metric	Value
Total confirmed cases	6,892
Vaccinated cases, n (%)	997 (14.5)
Unvaccinated cases, n (%)	5,895 (85.5)
Population vaccination coverage (PPV)*	90
Vaccine Effectiveness (95% CI)**	98.1 (98-98.2)
Relative Risk (unvaccinated vs vaccinated)	53.2
Population Attributable Fraction	97.8

*PPV = Population Proportion Vaccinated, estimated from mean MCV1 coverage 2014-2023

**VE calculated using Farrington screening method: VE = 1 - [(PCV x (1-PPV)) / (PPV x (1-PCV))]

Supplementary Table S11. Vaccine Effectiveness by state (states with >=20 cases)

State	Cases	Vaccinated, n (%)	PPV (%)	VE, % (95% CI)	Coverage 2023 (%)	Years <80%
Chihuahua	4,497	586 (13)	82.7	96.9 (96.6-97.1)	65.6	15
Jalisco	914	204 (22.3)	81.6	93.5 (92.4-94.4)	64.3	14
Chiapas	346	29 (8.4)	85.5	98.5 (97.7-98.9)	69.8	20
Guerrero	256	36 (14.1)	94.9	99.1 (98.8-99.4)	80.1	6
Michoacán	240	24 (10)	89.2	98.6 (97.9-99.1)	79.1	14
Sinaloa	122	29 (23.8)	91.0	96.9 (95.3-98)	73.8	4
Sonora	112	19 (17)	91.8	98.2 (97-98.9)	90.5	4
Ciudad de México	63	8 (12.7)	89.3	98.2 (96.3-99.2)	61.1	16
Colima	56	4 (7.1)	115.8	101 (NaN-NaN)	69.5	16
Coahuila	53	7 (13.2)	92.1	98.7 (97.1-99.4)	70.0	18
México	28	6 (21.4)	92.0	97.6 (94.2-99)	78.8	12
Morelos	27	0 (0)	90.0	100 (NaN-100)	77.2	11
Durango	26	3 (11.5)	89.6	98.5 (94.9-99.5)	83.5	11
Zacatecas	25	4 (16)	96.1	99.2 (97.8-99.7)	88.3	14
Baja California	20	9 (45)	84.1	84.5 (62.7-93.6)	63.5	15

PPV = Population Proportion Vaccinated (mean MCV1 coverage 2014-2023)
VE = Vaccine Effectiveness calculated using Farrington screening method
95% CI calculated using Orenstein method

Supplementary Table S12. Bivariable analysis of risk factors for measles complications, Mexico 2025-2026.

Variable	Exposed n/N (%)	Non-exposed n/N (%)	OR (95% CI)	p_value	Select p<0.20
Age <1 year	244/690 (35.4%)	825/6202 (13.3%)	3.57 (3-4.24)	<0.001	Yes
Age <5 years	550/1741 (31.6%)	519/5151 (10.1%)	4.12 (3.6-4.72)	<0.001	Yes
Female sex	524/3382 (15.5%)	545/3510 (15.5%)	1 (0.88-1.14)	0.970	
Speaks indigenous language	548/2003 (27.4%)	521/4889 (10.7%)	3.16 (2.76-3.61)	<0.001	Yes
Indigenous self-identification	548/2003 (27.4%)	521/4889 (10.7%)	3.16 (2.76-3.61)	<0.001	Yes
Unvaccinated	988/5895 (16.8%)	81/997 (8.1%)	2.28 (1.8-2.89)	<0.001	Yes
SSA institution	49/1643 (3%)	1020/5249 (19.4%)	0.13 (0.1-0.17)	<0.001	Yes
Autochthonous case	33/255 (12.9%)	1036/6637 (15.6%)	0.8 (0.55-1.17)	0.249	
Late outbreak phase (week >=15)	736/3275 (22.5%)	333/3617 (9.2%)	2.86 (2.49-3.29)	<0.001	Yes
Resurgence wave (Jan 2026)	63/741 (8.5%)	1006/6151 (16.4%)	0.48 (0.36-0.62)	<0.001	Yes
Rural municipality (>50% in <5000 pop)	401/1460 (27.5%)	668/5429 (12.3%)	2.7 (2.34-3.11)	<0.001	Yes
High illiteracy (Q4)	351/1656 (21.2%)	718/5233 (13.7%)	1.69 (1.47-1.95)	<0.001	Yes
High overcrowding (Q4)	350/1699 (20.6%)	719/5190 (13.9%)	1.61 (1.4-1.86)	<0.001	Yes
Without piped water (Q4)	384/1721 (22.3%)	685/5168 (13.3%)	1.88 (1.64-2.16)	<0.001	Yes
Without drainage (Q4)	423/1717 (24.6%)	646/5172 (12.5%)	2.29 (2-2.63)	<0.001	Yes
High poverty - income <2MW (Q4)	368/1716 (21.4%)	701/5173 (13.6%)	1.74 (1.51-2)	<0.001	Yes
Without health services (Q4)	153/1722 (8.9%)	916/5167 (17.7%)	0.45 (0.38-0.54)	<0.001	Yes
High seasonal workers (T3)	237/2072 (11.4%)	825/4666 (17.7%)	0.6 (0.52-0.7)	<0.001	Yes
Age (per year)	-	-	0.948 (0.943-0.954)	<0.001	Yes
Marginalization index (per unit)	-	-	0.937 (0.929-0.945)	<0.001	Yes
Social lag index (per unit)	-	-	1.351 (1.298-1.406)	<0.001	Yes
% without health services (per 10%)	-	-	0.723 (0.67-0.78)	<0.001	Yes
% illiteracy (per 10%)	-	-	1.429 (1.343-1.52)	<0.001	Yes
% overcrowding (per 10%)	-	-	1.248 (1.181-1.319)	<0.001	Yes
% rural population (per 10%)	-	-	1.13 (1.109-1.151)	<0.001	Yes
% income <2MW (per 10%)	-	-	1.211 (1.161-1.263)	<0.001	Yes
% seasonal workers (per 10%)	-	-	0.745 (0.702-0.79)	<0.001	Yes

OR = Odds Ratio; CI = Confidence Interval
Q4 = Fourth quartile (highest 25%); T3 = Third tertile (highest 33%)
MW = Minimum wage; Variables with p<0.20 are candidates for multivariable model
Twenty-eight individual-level and municipal-level variables assessed using logistic regression. Categorical variables: exposed vs non-exposed groups shown with n/N (%) complications and unadjusted OR (95% CI). Continuous variables: OR per specified unit change. Variables with p < 0.20 were candidates for the multivariable model (Hosmer-Lemeshow approach). Dichotomous municipal indicators based on national quartile (Q4) or tertile (T3) cutoffs.

Supplementary Table S13. Hospital discharge characteristics of measles cases from Ministry of Health (SSA) facilities, Mexico 2025 (N = 663).

A. Distribution of complications among hospitalized cases

Complication Type	n	%
No inhospital complication	355	53.5
Pneumonia	270	40.7
Encephalitis	10	1.5
Otitis media	3	0.5
Other complications	18	2.7
Other diagnosis	7	1.1

B. Complications by age group

Age Group	Total n	Pneumonia n (%)	Encephalitis n (%)	Otitis media n (%)	Other n (%)	No complication n (%)
<1 year	6	1 (16.7)	0 (0)	0 (0)	0 (0)	5 (83.3)
1-4 years	240	115 (47.9)	2 (0.8)	0 (0)	8 (3.3)	115 (47.9)
5-9 years	160	79 (49.4)	5 (3.1)	1 (0.6)	5 (3.1)	70 (43.8)
10-19 years	115	50 (43.5)	1 (0.9)	0 (0)	4 (3.5)	60 (52.2)
20-39 years	108	17 (15.7)	2 (1.9)	2 (1.9)	6 (5.6)	81 (75)
>=40 years	34	8 (23.5)	0 (0)	0 (0)	2 (5.9)	24 (70.6)

C. Length of hospital stay by complication type

Complication	n	Median (IQR) days	Mean (SD) days	Range
No complication	355	4 (2-6)	5.4 (11.3)	0-180
Pneumonia	270	4 (3-6)	6.2 (13.1)	1-132
Encephalitis	10	3.5 (3-4.75)	3.7 (1.6)	1-6
Otitis media	3	4 (4-7)	6 (3.5)	4-10
Other complications	18	3.5 (2.25-5.5)	4.3 (2.5)	2-10
Other diagnosis	7	6 (4-6.5)	5.4 (2.8)	1-10

D. Hospital outcomes by indigenous status

Variable	Indigenous (n=370)	Non-indigenous	p-value
N	370	293	
Length of stay, median (IQR)	4 (3-6)	4 (2-5)	<0.001
With complications, n (%)	186 (50.3)	122 (41.6)	0.033
Pneumonia, n (%)	169 (45.7)	101 (34.5)	0.005

[1]Mann-Whitney U test; [2]Chi-squared test; [3]Fisher's exact test. Analysis of the Automated Hospital Discharge Subsystem (SAEH) for measles-coded hospitalizations (ICD-10 B05x), February-November 2025. Panel A: Distribution of complications; Panel B: Complications by age group; Panel C: Length of hospital stay by complication type; Panel D: Hospital outcomes by indigenous status. Data cutoff: December 1, 2025.

Note: SAEH captures hospitalizations from Ministry of Health (Secretaría de Salud) facilities only. Hospitalizations in IMSS, ISSSTE, and private facilities are not included.

Supplementary Note S1. Estimation of the Effective Reproduction Number (Rt)

Theoretical Framework

The instantaneous effective reproduction number (Rt) represents the average number of secondary cases generated by a single infected individual at time t, given the current level of immunity and interventions in the population. Unlike the basic reproduction number (R0), Rt varies over time and reflects real-time transmission dynamics.

Rt was estimated using the framework proposed by Cori et al. (2013), implemented in the EpiEstim package (v2.2) for R. This method models Rt as the ratio of current incidence to the total infectiousness of previously infected individuals, weighted by the serial interval distribution.

Mathematical Formulation

Instantaneous Reproduction Number

$$R_t = I_t / \Lambda_t$$

Where:
I_t = number of new cases at time t
Λ_t = total infectiousness at time t

$$\Lambda_t = \Sigma_{s=1}^{t} I_{t-s} \cdot w_s$$

Bayesian Estimation

$$\text{Prior:} \quad R_t \sim \text{Gamma}(a, b)$$
$$\text{Posterior:} \quad R_t \mid I_t \sim \text{Gamma}(a + \Sigma I_s, 1 / (1/b + \Sigma \Lambda_s))$$

Posterior mean:
$$E[R_t] = (a + \Sigma I_s) / (1/b + \Sigma \Lambda_s)$$

A 7-day sliding window ($\tau = 7$) was used:
$$R_t^\tau = (a + \Sigma_{t-\tau+1}^{t} I_s) / (1/b + \Sigma_{t-\tau+1}^{t} \Lambda_s)$$

Serial Interval Parameters

The serial interval was modeled using a Gamma distribution based on Lessler et al. (2009):
Mean = 11.7 days
SD = 2.0 days
Shape = 34.2
Rate = 2.925

Minimum Incidence Filter

If $\Sigma_{t-6}^{t} I_s < 5$, then R_t was suppressed (set to NA).

Analytical Scope

Rt was estimated at national and state levels using daily incidence data derived from FECHA_DIAGNOSTICO.

S1.6 Interpretation

Rt > 1 indicates epidemic growth.
Rt = 1 indicates stable transmission.
Rt < 1 indicates epidemic decline.

Software and Reproducibility

R version 4.5.2
EpiEstim v2.2
Gamma(a=1, b=5)
7-day sliding window
